# Nanoscale Metal–Organic Framework Confines Zinc-Phthalocyanine Photosensitizers for Enhanced Photodynamic Therapy

**DOI:** 10.1021/jacs.1c07379

**Published:** 2021-08-23

**Authors:** Taokun Luo, Geoffrey T. Nash, Ziwan Xu, Xiaomin Jiang, Jianqiao Liu, Wenbin Lin

**Affiliations:** † Department of Chemistry, The University of Chicago, Chicago, Illinois 60637, United States; ‡ Department of Radiation and Cellular Oncology and Ludwig Center for Metastasis Research, The University of Chicago, Chicago, Illinois 60637, United States

## Abstract

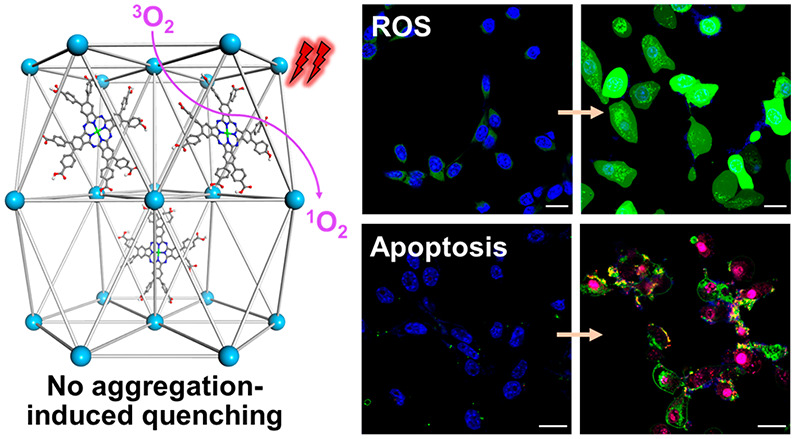

The performance of photodynamic therapy (PDT) depends on the solubility, pharmacokinetic behaviors, and photophysical properties of photosensitizers (PSs). However, highly conjugated PSs with strong reactive oxygen species (ROS) generation efficiency tend to have poor solubility and aggregate in aqueous environments, leading to suboptimal PDT performance. Here, we report a new strategy to load highly conjugated but poorly soluble zinc-phthalocyanine (ZnP) PSs in the pores of a Hf_12_-QC (QC = 2″,3′-dinitro-[1,1’:4′,1”;4″,1’”-quaterphenyl]-4,4’”-dicarboxylate) nanoscale metal–organic framework to afford ZnP@Hf-QC with spatially confined ZnP PSs. ZnP@Hf-QC avoids aggregation-induced quenching of ZnP excited states to significantly enhance ROS generation upon light irradiation. With higher cellular uptake, enhanced ROS generation, and better biocompatibility, ZnP@Hf-QC mediated PDT exhibited an IC_50_ of 0.14 μM and achieved exceptional antitumor efficacy with >99% tumor growth inhibition and 80% cure rates on two murine colon cancer models.

Photodynamic therapy (PDT) destroys a malignant tumor while sparing surrounding normal tissues by localizing a photosensitizer (PS) in the tumor and irradiating the tumor with visible or near-infrared light to produce cytotoxic reactive oxygen species (ROS).^1−4^ The clinical utility of PDT is limited by tissue penetration of light, localization of the PS in the tumor, and the solubility and photophysical properties of the PS.^5,6^ For example, clinically used porphyrin-based PSs often cause phototoxicity side effects due to their strong absorption in the visible spectrum and retention in the skin.^7^ Phthalocyanine (Pc) PSs present a promising alternative due to their very strong absorption in 650–800 nm and weak absorption in 400–600 nm, allowing for effective treatment of tumors with low PS doses and reduced phototoxicity.^8^ Metalation of Pcs with diamagnetic ions (e.g., Zn^2+^, Si^4+^, Al^3+^) increases triplet state yields and lifetimes to enhance the generation of cytotoxic singlet oxygen (^1^O_2_).^9−11^

Despite their improved photophysical properties, Pcs have not been widely used for PDT due to their limited synthetic accessibility and their strong tendency to aggregate in biological media.^11^ Pcs have been functionalized with ionic or hydrophilic groups in their peripheral positions to increase aqueous solubility or coordinate with bulky metal complexes (axial functionalization) to prevent π–π stacking.^12^ However, the introduction of ionic or hydrophilic groups to Pcs can adversely impact their cellular uptake while axial functionalization of Pcs is limited to a few nontoxic high-valent metals such as Si^4+^.^13,14^

An alternative strategy to address the solubility and aggregation issues of Pcs is through their encapsulation in or conjugation to liposomes, micelles, or other nanoparticles (NPs).^15−19^ In particular, micelles have been widely investigated as a delivery vehicle for lipophilic conjugated Pcs with superb photophysical properties.^20,21^ Nanoscale metal–organic frameworks (nMOFs) have recently provided an excellent strategy to deliver porphyrin, chlorin, and bacteriochlorin PSs for PDT.^22−28^ With structural tunability, rigidity, and porosity, nMOFs can efficiently load PSs via direct incorporation as bridging ligands, postsynthetic ligand exchange, postsynthetic surface modification, and physical loading into pores.^29−35^ These strategies allow isolation or confinement of lipophilic PSs in rigid nMOF structures to reduce aggregation, improve cellular uptake, and reduce photodegradation.^36−41^ We hypothesized that nMOFs could also be used to encapsulate Pcs to enhance their PDT efficacy.

Herein, we report the design of a Hf-QC nMOF based on Hf_12_ secondary building units (SBUs) and QC bridging ligands (QC = 2″,3′-dinitro-[1,1’:4′,1”;4″,1’”-quaterphenyl]-4,4’”-dicarboxylate) for the delivery of zinc(II)-2,3,9,10,16,17,23,24-octa(4-carboxyphenyl)-phthalocyanine (ZnP) PSs for highly efficient type II PDT (Figure 1).^42^ Postsynthetic loading of ZnP into the pores of the rigid Hf-QC framework afforded ZnP@Hf-QC. The confined PSs in ZnP@Hf-QC efficiently absorbed light and avoided aggregation-induced quenching to significantly enhance ^1^O_2_ generation and effectively eradicated/regressed colorectal cancer in mouse models.

**Figure 1:**
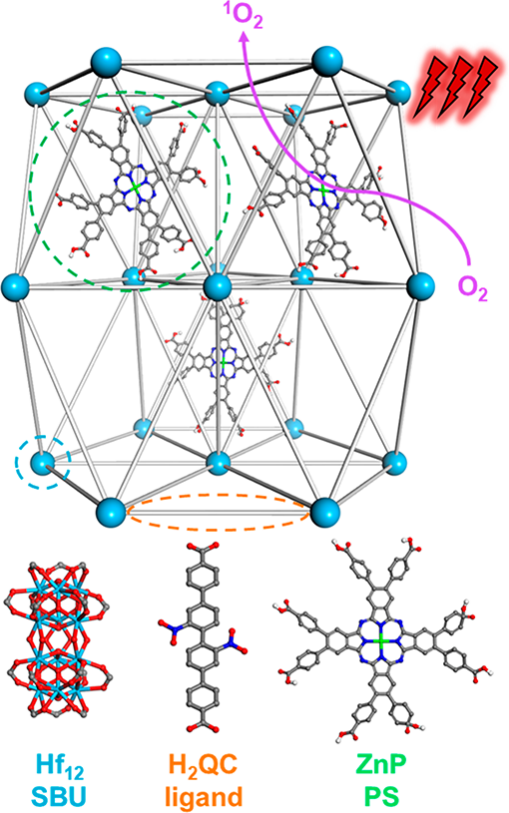
Scheme showing the structure of ZnP@Hf-QC consisting of a 3D framework of Hf_12_ SBUs and QC bridging ligands and ZnP PSs confined in the pores. ZnP@Hf-QC efficiently generates ^1^O_2_ upon 700 nm light irradiation.

Hf-QC was synthesized through a solvothermal reaction between HfCl_4_ and H_2_QC in a mixture of *N,N*-dimethylformamide (DMF), acetic acid, and water at 80 °C (Figure 2a). Transmission electron microscopy (TEM) imaging of Hf-QC revealed a hexagonal nanoplate morphology with a diameter of ∼150 nm while atomic force microscopy (AFM) showed a plate thickness of ∼64 nm (Figure 2b,d). Dynamic light scattering (DLS) measurements of Hf-QC gave a number-averaged size of 167.1 ± 2.9 nm (Figure 3c). Powder X-ray diffraction (PXRD) studies (Figure 3b) showed that Hf-QC adopted the same **hcp** topology as previously reported Zr_12_-QPDC (QPDC = para-quaterphenyldicarboxylate).^43^ High resolution TEM (HRTEM) imaging and fast Fourier transform (FFT) pattern of Hf-QC revealed a lattice point distance of 2.3 nm and displayed a 6-fold symmetry (Figure 2c, Figure S15), which matched well with the modeled structure for Zr_12_-QPDC. ^1^H nuclear magnetic resonance (NMR) analysis of digested Hf-QC showed an acetate (OAc) modulator to QC linker ratio of 0.11:1, corresponding to approximately 0.5 missing linkers per SBU (Figure S11). Thermogravimetric analysis (TGA) of Hf-QC showed a weight loss of 39.4% in the 300–800 °C region, matching the expected value of 37.9% for the Hf-QC with a 0.5 linker defect per SBU (Figure S14). On the basis of these results, Hf-QC was formulated as Hf_12_(μ_3_-O)_8_(μ_3_–OH)_8_(μ_2_–OH)_6_(QC)_8.5_(OAc).

**Figure 2:**
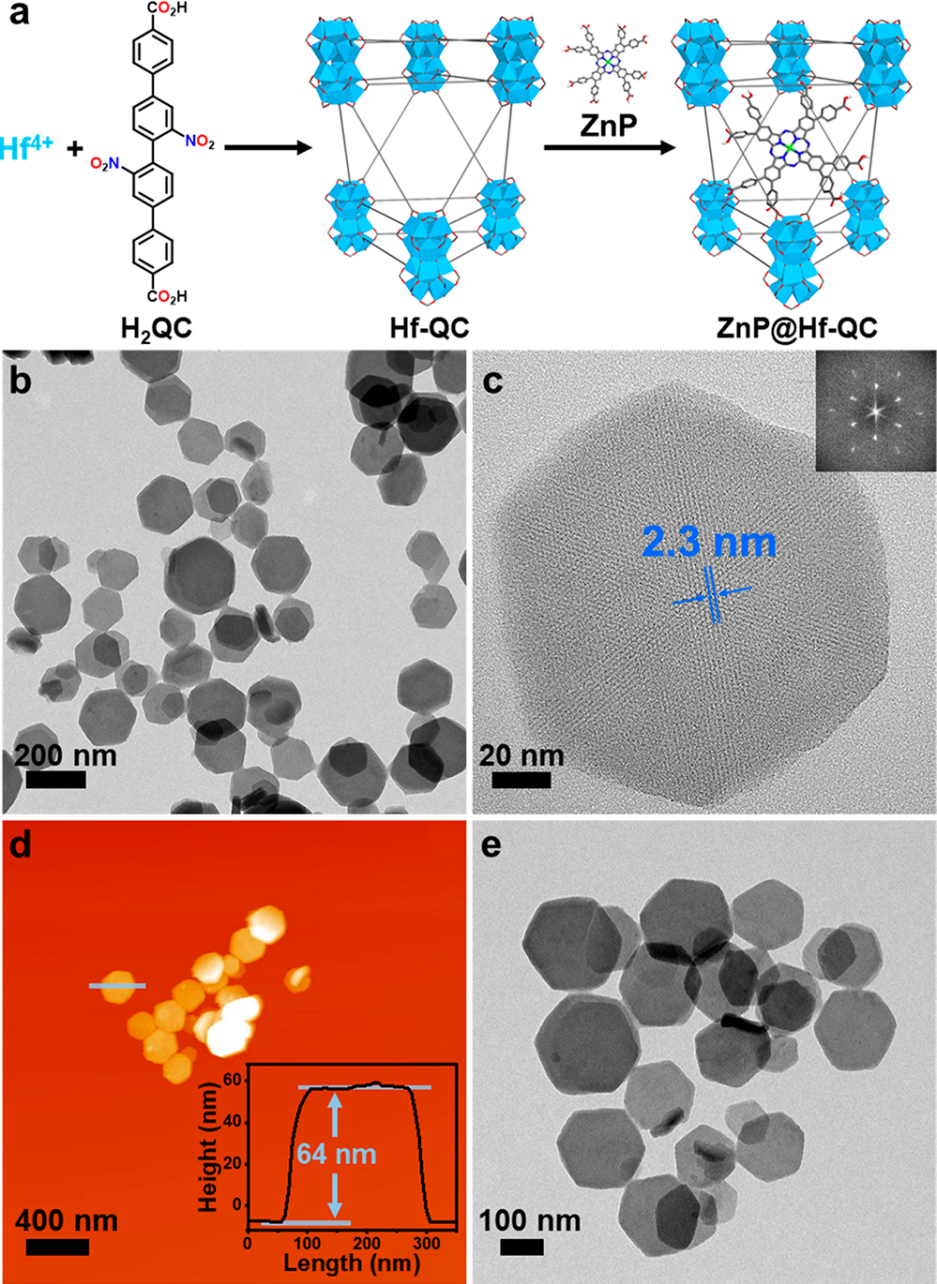
(a) Synthetic scheme of ZnP@Hf-QC. (b) TEM image and (c) HRTEM image and its FFT pattern (inset) of Hf-QC. (d) AFM topography and height profile (inset) of Hf-QC. (e) TEM image of ZnP@Hf-QC.

**Figure 3:**
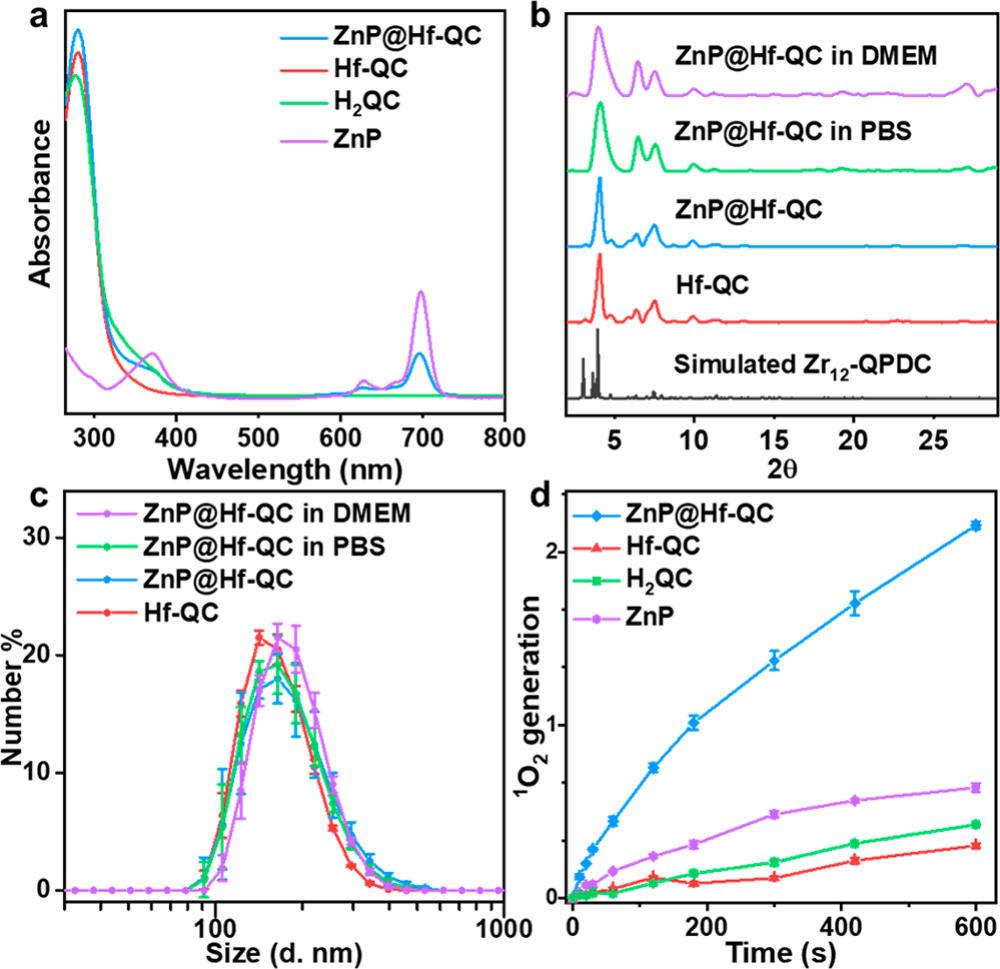
(a) UV–vis spectra of ZnP, H_2_QC, Hf-QC, and ZnP@Hf-QC in DMSO. (b) PXRD patterns of Hf-QC, ZnP@Hf-QC (as synthesized and after soaking in PBS or DMEM for 24 h), and the simulated pattern for Zr_12_-QPDC. (c) Number-averaged diameters of Hf-QC and ZnP@Hf-QC (as synthesized and after soaking in PBS or DMEM for 24 h) in ethanol. (d) ^1^O_2_ generation of ZnP, H_2_QC, Hf-QC, and ZnP@Hf-QC detected by SOSG assay (*n* = 3).

ZnP@Hf-QC was synthesized by heating a mixture of ZnP and Hf-QC in DMF at 70 °C for 24 h. Loading of ZnP in ZnP@Hf-QC was confirmed by the presence of characteristic Ultraviolet–visible (UV–vis) and infrared (IR) peaks for ZnP (Figure 3a, Figure S18). UV–vis spectroscopy and inductively coupled plasma-mass spectrometry (ICP-MS) showed the loading of 13.6 wt % ZnP in ZnP@Hf-QC, corresponding to a ZnP to Hf_12_ SBU ratio of 0.68:1. ^1^H NMR analysis of digested ZnP@Hf-QC showed that the OAc modulator to QC linker ratio was maintained after ZnP loading (Figure S19). TGA of ZnP@Hf-QC showed a weight loss of 36.3% in the 300–800 °C region, which matched well with the expected value of 34.1% for physical loading of ZnP in the nMOF pores and confirmed the ratio of ZnP to Hf (Figure S14). On the basis of these results, ZnP@Hf-QC was formulated as (ZnP)_0.68_@Hf_12_(μ_3_-O)_8_(μ_3_–OH)_8_(μ_2_–OH)_6_(QC)_8.5_(OAc).

TEM and DLS showed that ZnP@Hf-QC retained the hexagonal nanoplate morphology and size (175.8 ± 5.6 nm) of Hf-QC (Figures 2e and 3c, Figure S15). HRTEM images (Figure S15) and PXRD patterns (Figure 3b) of ZnP@Hf-QC supported the maintenance of Hf-QC structure after ZnP loading. ZnP@Hf-QC displayed a slightly more negative ζ potential of −24.0 ± 1.5 mV compared to Hf-QC at −22.1 ± 0.7 mV (Figure S16), consistent with loading negatively charged ZnP into the pores of Hf-QC. The presence of QC and ZnP was confirmed by its characteristic UV–vis and ^1^H NMR signals in digested ZnP@Hf-QC (Figures S11, S19). The stability of ZnP@Hf-QC was demonstrated by PXRD and DLS after incubation in phosphate-buffered saline (PBS) or Dulbecco’s Modified Eagle Medium (DMEM) at 37 °C for 24 h (Figure 3b,c).

ZnP@Hf-QC showed a much higher cellular uptake than free ZnP and accumulated in endo/lysosomes. Confocal laser scanning microscopy (CLSM) revealed that fluorescence signals of ZnP@Hf-QC started to overlap with endo/lysosomes in CT26 cells after incubation for 12 h (Figure 4b–f, Figure S23).^44^ However, fluorescence signals were barely observed for CT26 cells incubated with free ZnP (Figure 4a, Figure S22). Quantification of cellular uptake by UV–vis spectroscopy showed that ZnP@Hf-QC delivered up to15-fold more ZnP than free ZnP *in vitro* (Figure 5d).

**Figure 4:**
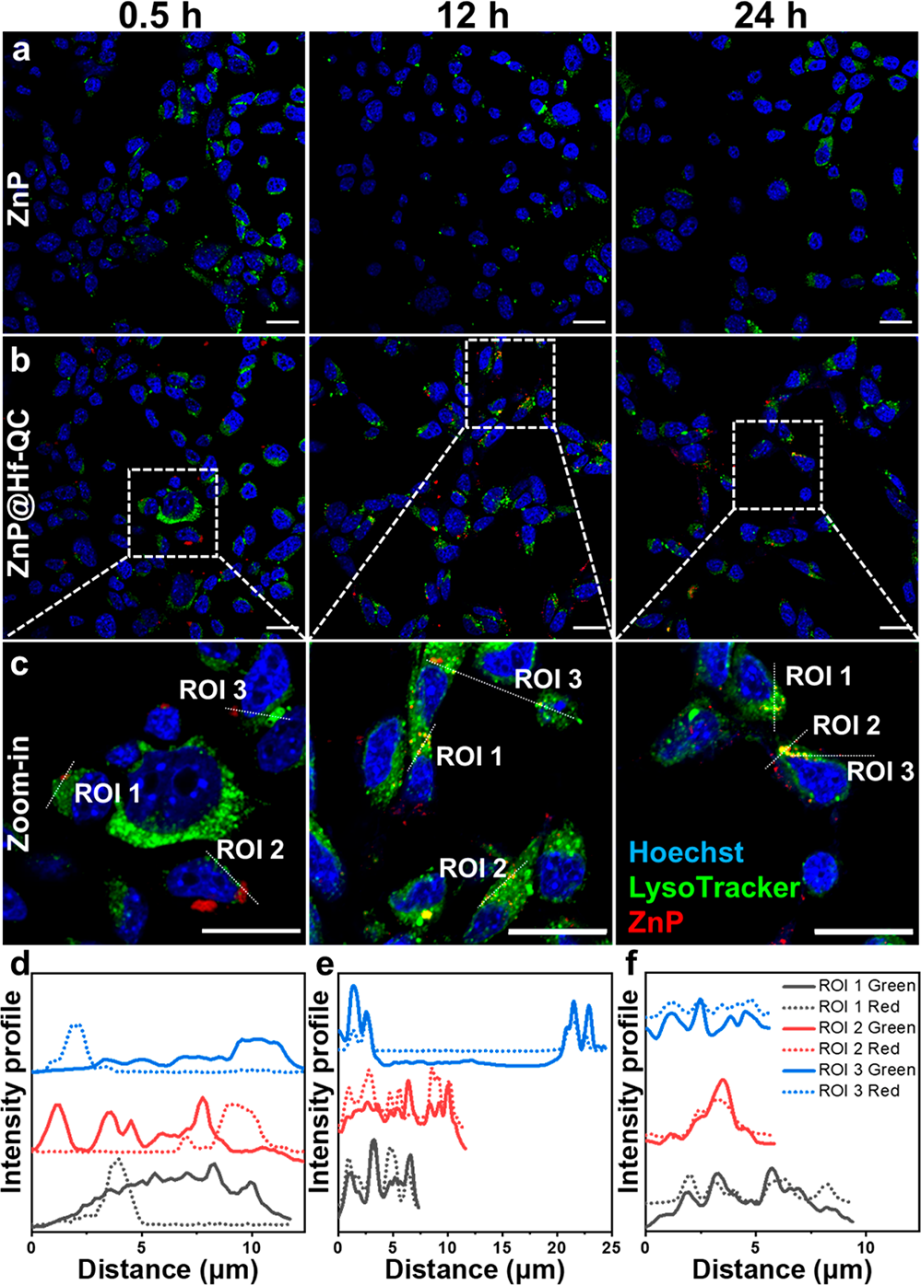
CLSM images showing colocalization of ZnP (a) and ZnP@Hf-QC (b, c) with endo/lysosomes after incubation with CT26 cells for 0.5, 12, and 24 h (yellow = green + red, scale bars are 20 μm). (d–f) Colocalization analysis between endo/lysosomes (green) and ZnP (red) in different ROIs (white dashed lines in Figure 4c).

**Figure 5:**
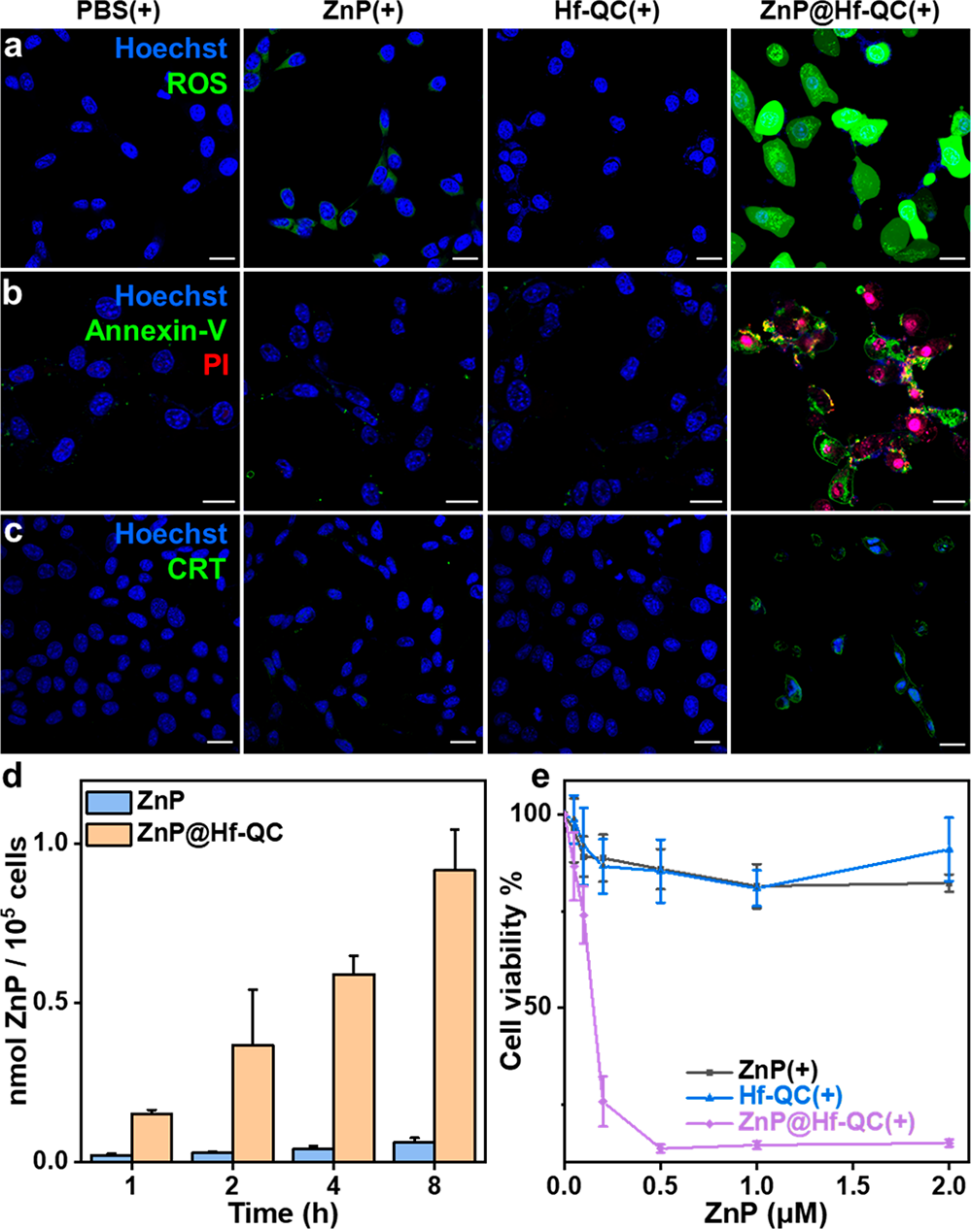
(a) Total ROS generation by DCF-DA assay (green). (b) Cell apoptosis stained by Alexa Fluor 488 Annexin V (green) and PI (red) (pink = red + blue). (c) CRT translocation stained by Alexa Fluor 488 CRT antibody (green) (d) Cellular uptake measured by UV–vis spectroscopy, *n* = 3. (e) Viability of cells treated with ZnP(+), Hf-QC(+), and ZnP@Hf-QC(+) by MTS assay. Cell nuclei were stained by Hoechst 33342 (blue) in (a-c). CT26 cells were used for all *in vitro* experiments and a total light dose of 60 J/cm^2^ was given. All scale bars equal 20 μm.

^1^O_2_ generation by ZnP and ZnP@Hf-QC was determined by singlet oxygen sensor green (SOSG) assay. ZnP@Hf-QC generated 3.4-fold as much ^1^O_2_ as free ZnP (Figure 3d), indicating that the entrapment of ZnP PSs in MOF pores prevented aggregation-induced quenching of ZnP excited states and enhanced ^1^O_2_ generation in a type II PDT process. CLSM imaging (Figure 5a, Figure S29) and flow cytometry analysis (Figure S28) showed a ROS burst in CT26 cells incubated with ZnP@Hf-QC after light irradiation (denoted as “+”, 100 mW/cm^2^, 10 min, “-” denotes no light treatment) by 2′,7′-dichlorodihydrofluorescein diacetate (DCF-DA) assay, confirming the enhanced ROS generation by ZnP@Hf-QC *in vitro*. MTS assays showed that ZnP(+) exhibited minimal toxicity at concentrations up to 2 μM, while ZnP@Hf-QC(+) was highly cytotoxic with an IC_50_ of 0.14 μM (Figure 5e, Figure S24). No obvious toxicity or morphological changes were observed for CT26 cells treated with Hf-QC(−), Hf-QC(+), or ZnP@Hf-QC(−). Live cell imaging confirmed significant growth inhibition of CT26 cells by ZnP@Hf-QC(+) (Figure S25, Movies S1, S2).

We then examined apoptosis and immunogenic cell death of CT26 cells after PDT by CLSM and flow cytometry. CT26 cells treated with ZnP@Hf-QC(+) showed upregulation of phosphatidylserine by Annexin V staining on cell membranes and colocalization of propidium iodine (PI) and Hoechst 33342 (Figure 5b, Figures S26, S27). These results indicated apoptosis and compromised membrane functions for ZnP@Hf-QC(+) treated CT26 cells, which were absent in control groups. Calreticulin (CRT) staining revealed enhanced immunogenic cell death and surface translocation of CRT signals in ZnP@Hf-QC(+) group (Figure 5c, Figures S30, S31). Taken together, ZnP@Hf-QC(+) not only killed cancer cells more effectively but also induced immunogenic cell death to expose tumor antigens and danger signals for immune activation.^45,46^

We evaluated antitumor efficacy of ZnP@Hf-QC(+) on two subcutaneous murine colon cancer models with CT26 tumors on BALB/c mice and MC38 tumors on C57BL/6 mice. Hf-QC and ZnP@Hf-QC were pegylated before intravenous administration. The mice were injected with PBS, ZnP, Hf-QC, or ZnP@Hf-QC via tail veins at an equivalent ZnP dose of 50 nmol (equivalent Hf dose of 0.88 μmol). Twelve hours post injection, the mice were anesthetized, and tumor areas were irradiated with 700 nm LED with a total light flux of 60 J/cm^2^ (100 mW/cm^2^).

Compared to PBS(+), Hf-QC(+) had little effect on tumor growth with minimal tumor growth inhibition indices (TGIs) of 17.8% and 7.4% for CT26 and MC38 tumors, respectively. ZnP(+) moderately slowed tumor growth with TGI values of 41.3% and 41.4% for CT26 and MC38 tumors, respectively. ZnP@Hf-QC(+) treatment showed excellent antitumor efficacy with >99% TGIs and 80% cure rates for both CT26 and MC38 tumors (Figure 6a,b, Figures S32, S33, S38, S39). H&E and TUNEL staining revealed severe apoptosis/necrosis and infiltration of inflammatory cells in tumor regions in the ZnP@Hf-QC(+) group (Figure 6c, Figure S43). Several mice in the ZnP(+) and ZnP(−) groups showed weight loss, pulmonary edema, and local liver inflammation (Figures S35, S36), likely caused by aggregation of ZnP into large particles *in vivo*. In comparison, although ZnP@Hf-QC were observed to accumulate in spleens and livers similar to other nanoparticles^47,48^ (Figures S36, S37) by tumor tissue sections, mice treated with ZnP@Hf-QC with or without light irradiation showed steady body weights (Figures S41, S42). ZnP@Hf-QC and its aggregate were not observed in lungs and minimal abnormities were observed in the major organs of ZnP@Hf-QC treated mice compared to PBS control (Figures S34, S40). The different *in vivo* behaviors between ZnP and ZnP@Hf-QC showed that the nMOF pore loading strategy provides an efficient, safe, and biocompatible approach to deliver PSs with unfavorable solubility and pharmacokinetic properties.

**Figure 6:**
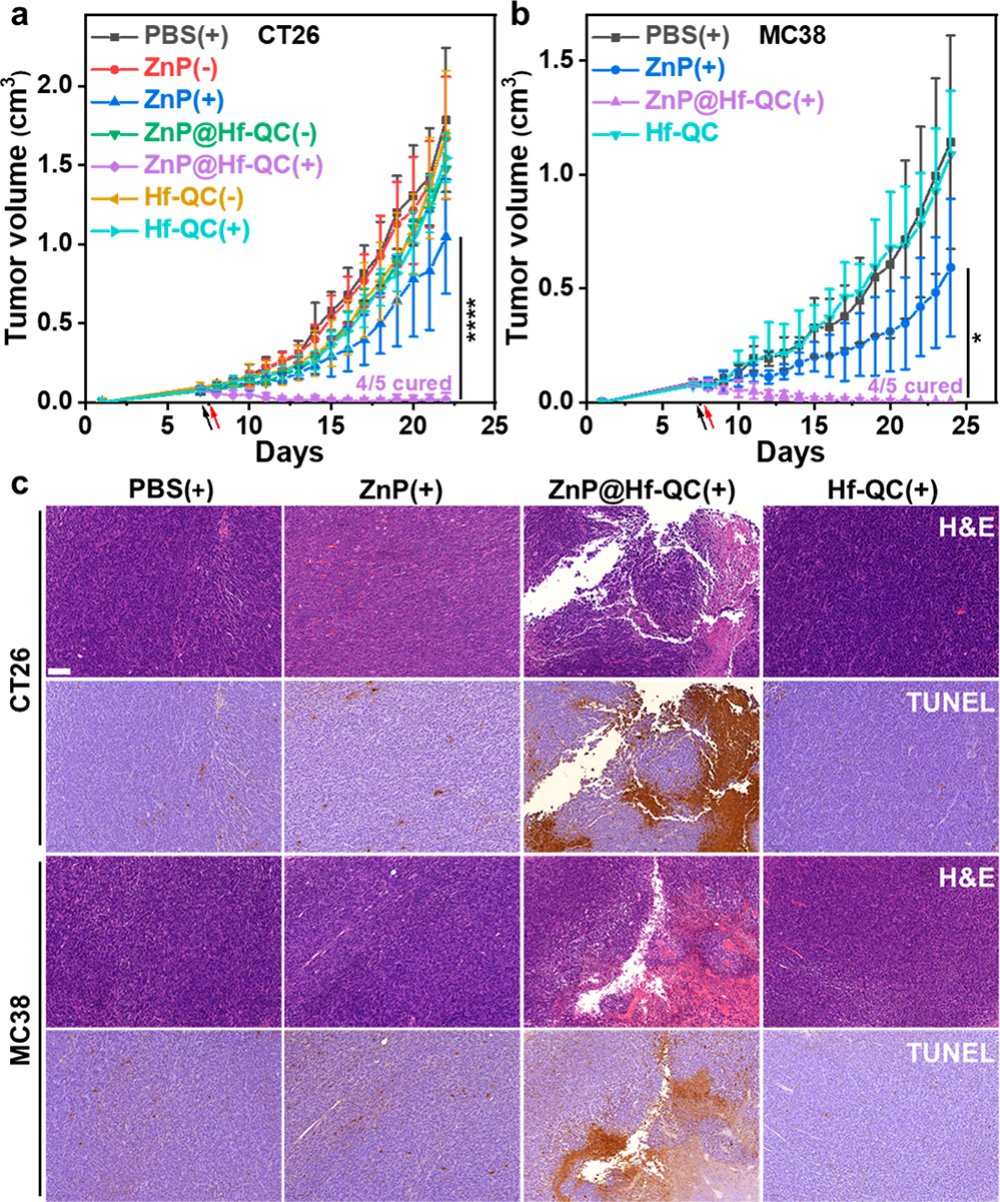
Antitumor efficacy of ZnP@Hf-QC(+) on subcutaneous CT26 tumor-bearing BALB/c (a) and MC38 tumor-bearing C57BL/6 (b) mouse models, *n* = 5. (c) Representative images of H&E staining and TUNEL IHC staining of excised CT26 and MC38 tumors. Scale bars are 100 μm, *p < 0.05 and ****p < 0.0001 by ANOVA with Tukey’s tests.

In summary, we developed an nMOF confinement strategy to isolate ZnP PSs and prevent their aggregation and excited state quenching. As a result, the isolated PSs in ZnP@Hf-QC efficiently absorbed light to significantly enhance ^1^O_2_ generation and efficiently kill cancer cells. ZnP@Hf-QC mediated PDT effectively eradicated/regressed colorectal cancers in two mouse models. The confinement of photosensitizers in nMOF pores provides a new strategy to unleash the potential of poorly soluble, highly conjugated PSs in PDT.

## Supporting Information Available

Synthesis and characterization of ZnP@Hf-QC, ROS generation, *in vitro* and *in vivo* anticancer efficacy studies (PDF)
Live imaging of cells treated by ZnP@Hf-QC mediated PDT (MP4)
(MP4)
