# Supplementary material for: Nanoscale Metal–Organic Framework Confines Zinc-Phthalocyanine Photosensitizers for Enhanced Photodynamic Therapy
Source: J Am Chem Soc. 2021 Aug 23;143(34):13519–24. doi: 10.1021/jacs.1c07379 (PMC8414475; doi:10.1021/jacs.1c07379)
Supplement: Supplementary file 1 — ja1c07379_si_001.pdf [file ja1c07379_si_001.pdf]

Supporting Information for

Nanoscale Metal-Organic Framework Confines Zinc-Phthalocyanine Photosensitizers for Enhanced Photodynamic Therapy

Taokun Luo,[1,‡] Geoffrey T. Nash,[1,‡] Ziwan Xu,[1] Xiaomin Jiang,[1] Jianqiao Liu,[1] and Wenbin Lin[1,2,*]

[1]Department of Chemistry, The University of Chicago, Chicago, Illinois 60637, United States

[2]Department of Radiation and Cellular Oncology and Ludwig Center for Metastasis Research, The University of Chicago, Chicago, Illinois 60637, United States

Email: wenbinlin@uchicago.edu

Table of Contents

S1. Materials and Methods 2
S2. Synthesis and Characterization of ZnP@Hf-QC 3
S3. Reactive Oxygen Species Generation 144
S4. *In vitro* study 144
S5. *In vivo* study 255
S6. References 355

S1. Materials and Methods

All starting materials were purchased from Sigma-Aldrich and Thermo Fisher Scientific (USA) unless otherwise noted and used without further purification. Transmission electron microscopy (TEM) was carried out on a TECNAI Spirit and a TECNAI F30 HRTEM. Atomic force microscopy (AFM) images were taken on a Bruker Multimode 8-HR instrument. Powder X-ray diffraction (PXRD) data was collected on a Bruker D8 Venture diffractometer using a Cu Kα radiation source (λ = 1.54178 Å) and processed with PowderX software. UV-Vis spectra were collected using a Shimadzu UV-2600 UV-Vis spectrophotometer. Infrared (IR) spectra were collected between 400-4000 cm^{-1} on a Thermo Scientific Nicolet iS50 FT-IR spectrophotometer equipped with a built-in diamond attenuated total reflectance (ATR) accessory (for powder samples). Fluorescence emission spectra were obtained using a Shimadzu RF-5301PC spectrofluorophotometer. Dynamic light scattering (DLS) and ζ potential measurements were performed on a Malvern Zetasizer Nano ZS instrument. Inductively coupled plasma-mass spectrometry (ICP-MS) data were collected using an Agilent 7700x ICP-MS and analyzed using an ICP-MS Mass Hunter version B01.03. Samples were diluted in a 2% HNO_3 matrix and analyzed with ^{159}Tb and internal standards against a 10-point standard curve between 1 ppb and 500 ppb. The correlation was given R>0.999 for all analyses of interest. Data collection was performed in Spectrum Mode with three replicates per sample and 100 sweeps per replicate. ^{1}H NMR spectra were recorded on a Bruker NMR 400 DRX spectrometer at 400 MHz and referenced to the proton resonance resulting from incomplete deuteration of $CDCl_3$ (δ 7.26) or DMSO-d_6 (δ 2.50). Matrix-assisted laser desorption/ionization-time of flight high resolution mass spectrometry (MALDI-TOF HRMS) data were collected on a Bruker Ultraflextreme MALDI-TOF/TOF using positive ion mode. Thermogravimetric analysis (TGA) was performed in air using a Shimadzu TGA-50 equipped with an alumina crucible and heated at a rate of 1 °C per min. Flow cytometry data was collected on an LSR-Fortessa 4-15 (BD Biosciences, USA) and analyzed by FlowJo software (Tree Star, USA). Confocal laser scanning microscope images were collected on a Leica Stellaris 8 laser scanning confocal microscope. CLSM imaging was performed at the University of Chicago Integrated Light Microscopy Facility and analysis was done with LAS X (Leica) and ImageJ software (NIH, USA). Live cell imaging was recorded and analyzed by IncuCyte S3 with standard mode at Cellular Screening Center at the University of Chicago. The histological slides were scanned on a CRi Pannoramic SCAN 40x whole slide scanner by Integrated Light Microscopy Core in the University of Chicago and analyzed with the QuPath-0.2.3 software.[1]

DPBS (-Mg^{2+}, Ca^{2+}) were purchased from Thermo Fisher Scientific. 3-(4,5- dimethylthiazol-2-yl)-5-(3-carboxymethoxyphenyl)-2-(4-sulfo-phenyl)-2H-tetrazolium (MTS) assay was purchased from Promega (USA). Murine colorectal carcinoma CT26 and MC38 cells were obtained from the American Type Culture Collection (ATCC, Rockville, MD) and were cultured in RPMI-1640 (Corning, USA) (Gibco, USA) or DMEM medium supported with 10% heat-inactivated fetal bovine serum (56°C 30 min, VWR, USA), 100 U/ml penicillin G sodium and 100 μg/ml streptomycin sulphate in a humidified atmosphere containing 5% CO_2 at 37°C. Mycoplasma was tested for all cells before use by MycoAlert detection kit (Lonza Nottingham, Ltd.). C57BL/6 and BALB/c mice (6-8weeks) were obtained from Charles River Laboratories (USA). The study protocol was reviewed and approved by the Institutional Animal Care and Use Committee (IACUC)

at the University of Chicago. The Human Tissue Resource Center at the University of Chicago provided the histology related services for this study.

S2. Synthesis and Characterization of ZnP@Hf-QC

S2.1. Synthesis and characterization of ZnP

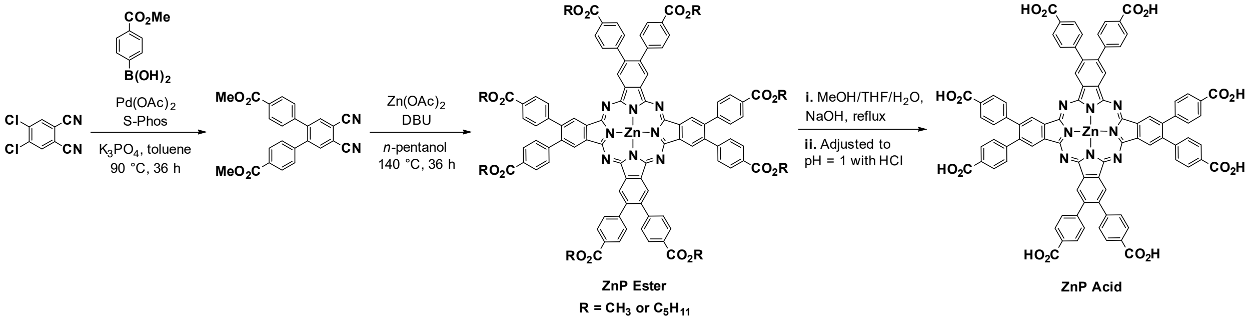

Figure S1. Synthesis of zinc(II)-2,3,9,10,16,17,23,24-octa(4-carboxyphenyl)-phthalocyanine (ZnP).

Synthesis of Zinc(II) 2,3,9,10,16,17,23,24-octa(4-carboxyphenyl)phthalocyanine (ZnP). ZnP was synthesized as shown in Figure S1 according to the previous literature report.[2] HRMS (MALDI-TOF) *m/z* Calcd. for $C_{88}H_{48}N_8O_{16}Zn^+$ ($[M^+]$) 1536.250, Found: 1536.145.

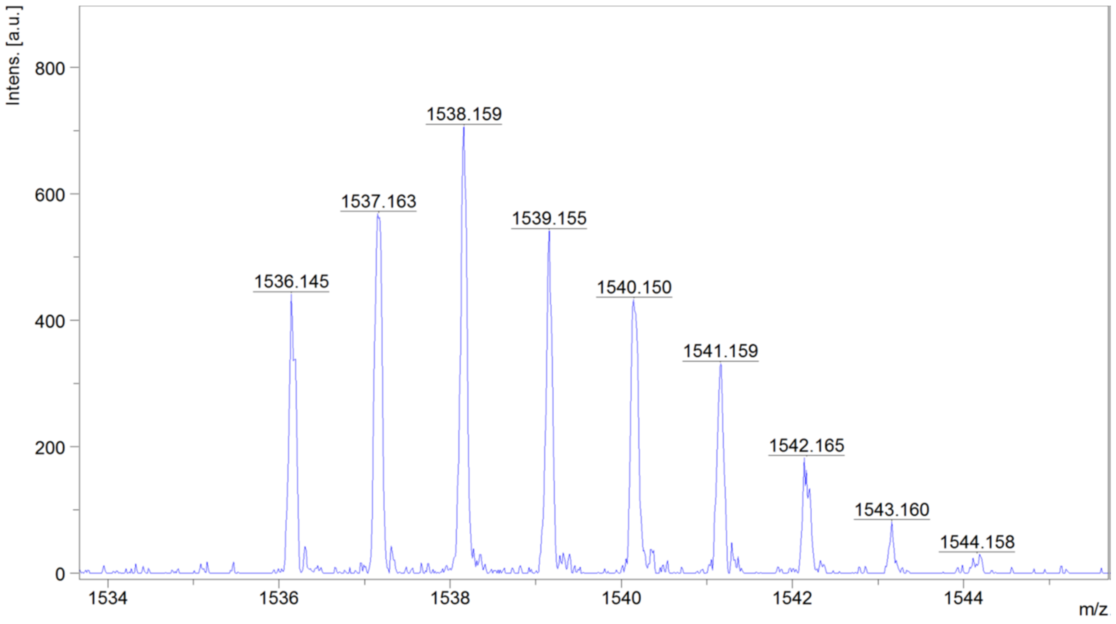

Figure S2. MALDI-TOF HRMS spectrum of ZnP showing isotopic peaks of the molecular ion.

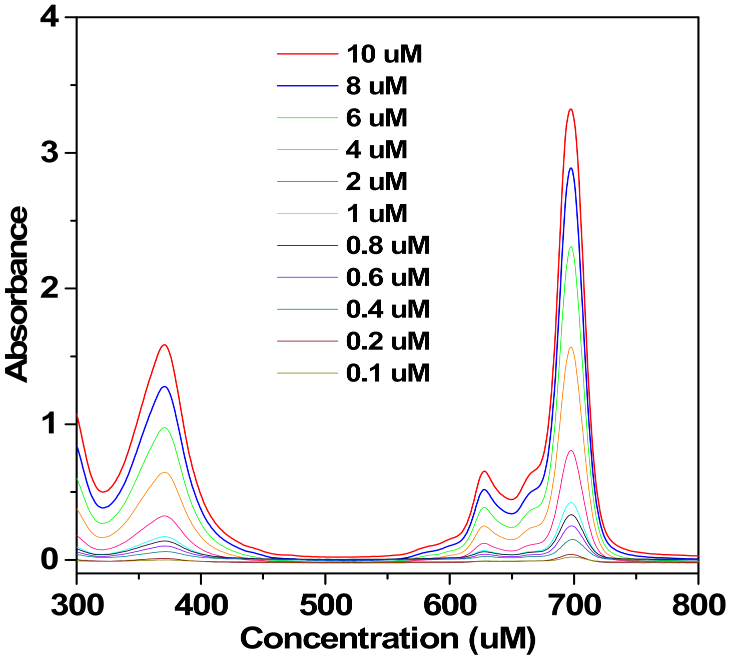

Figure S3. UV-Vis spectra of ZnP in DMSO at different concentrations.

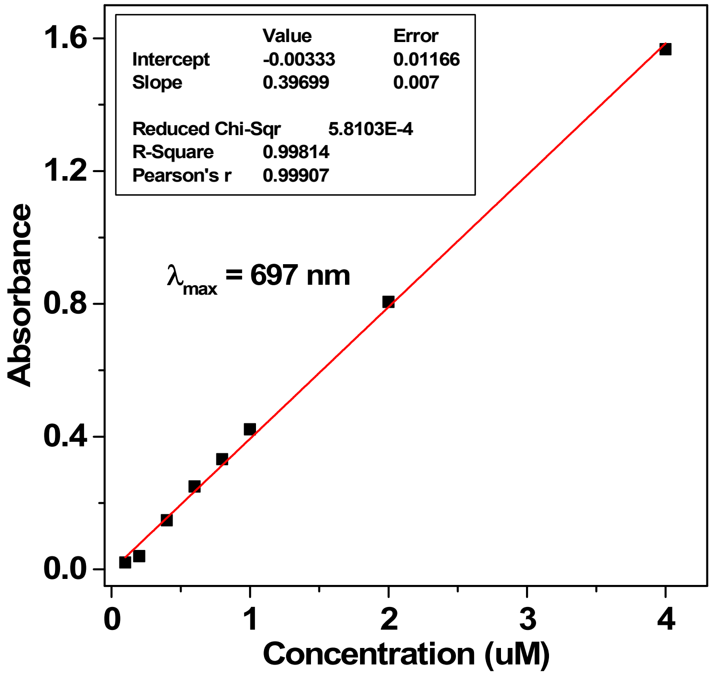

Figure S4. Plot of the absorbance of ZnP at 697 nm as a function of concentration.

2.2. Synthesis and characterization of Hf-QC

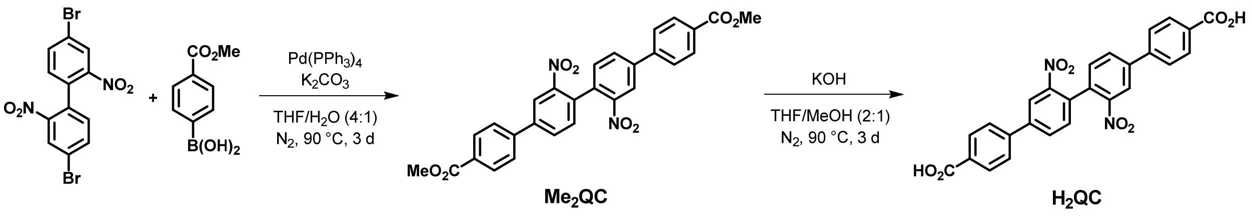

Figure S5. Synthesis of 2”,3’-dinitro-[1,1’:4’,1”;4”,1’”-quaterphenyl]-4,4’”-dicarboxylic acid (H_2QC).

Synthesis of Me_2QC. A mixture of $Pd(PPh_3)_4$ (231 mg, 0.2 mmol), K_2CO_3 (1.38 g, 10 mmol), 4,4’-dibromo-2,2’-dinitro-1,1’-biphenyl (804 mg, 2 mmol) and 4-methoxy-carbonylphenylboronic acid (1.08 g, 6 mmol) was dissolved in degassed THF/H_2O (40 mL/10 mL) in a 250 mL Schlenk tube. The resulting mixture was stirred under inert atmosphere at 90 °C for 3 days. The mixture was then cooled to room temperature, filtered, and washed with THF and ether to afford Me_2QC (568 mg, 1.11 mmol, 55% yield). ^{1}H NMR (500 MHz, DMSO-d_6): δ 8.59 (s, 2H), 8.27 (d, 2H), 8.13 (d, 4H), 8.06 (d, 4H), 7.70 (d, 2H), 3.91 (s, 6H).

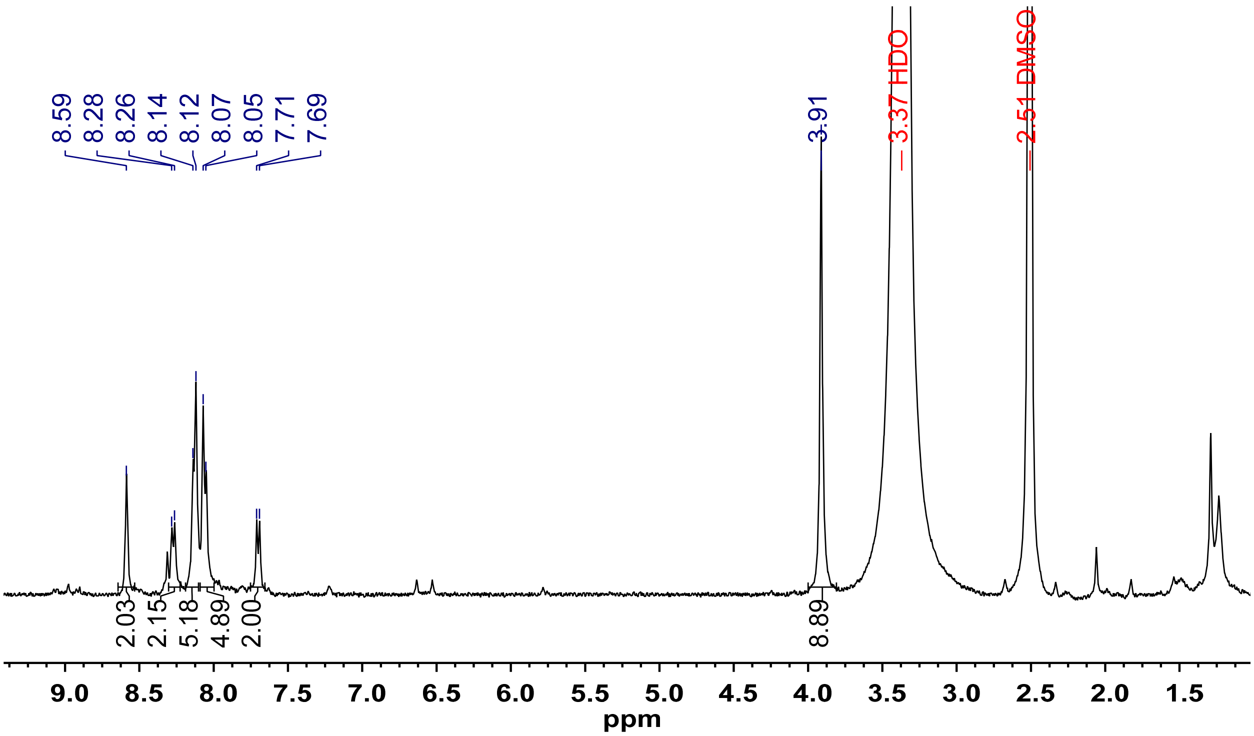

Figure S6. ^{1}H NMR spectrum of Me_2QC in DMSO-d_6.

Synthesis of H_2QC. Me_2QC (256 mg, 0.5 mmol) was suspended in 20 mL THF in a 50 mL round-bottomed flask followed by dropwise addition of a KOH/MeOH solution (2.8 g KOH in 10 mL MeOH). After the mixture was stirred at 40 C overnight, 1 M HCl (aq.) was slowly added until the pH was below 7. The solid was collected and washed with water, THF, and ether before being dried under vacuum to give the solid

(215 mg, 0.444 mmol, 89% yield). ^{1}H NMR (500 MHz, DMSO-d_6): δ 8.57 (s, 2H), 8.27 (d, 2H), 8.11 (d, 4H), 8.02 (d, 4H), 7.69 (d, 2H).

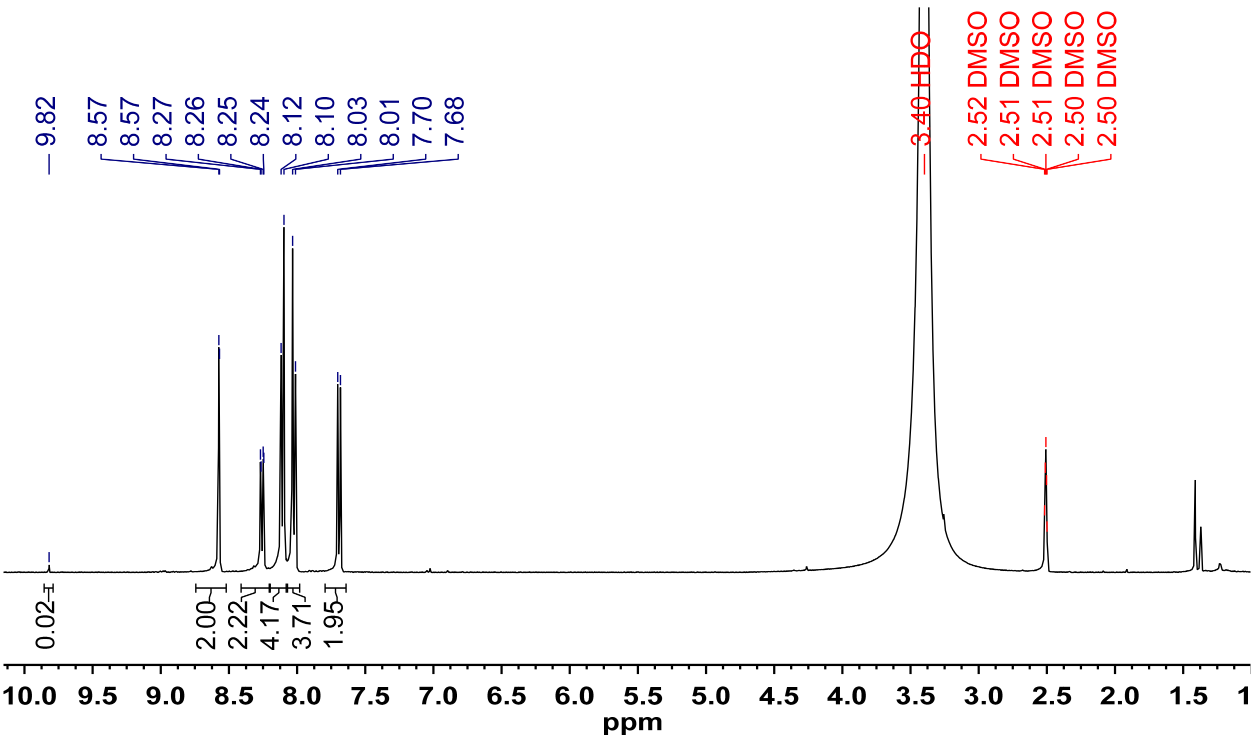

Figure S7. ^{1}H NMR spectrum of H_2QC in DMSO-d_6.

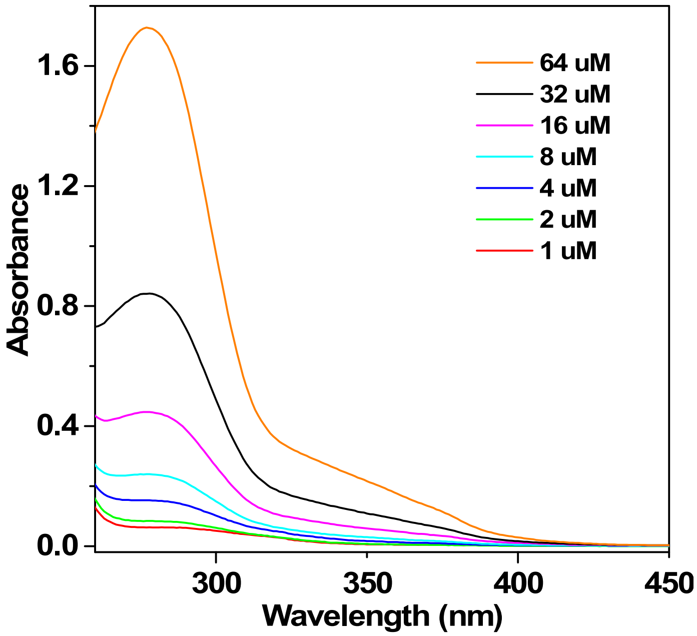

Figure S8. (a) UV-Vis spectra of H_2QC in DMSO at different concentrations.

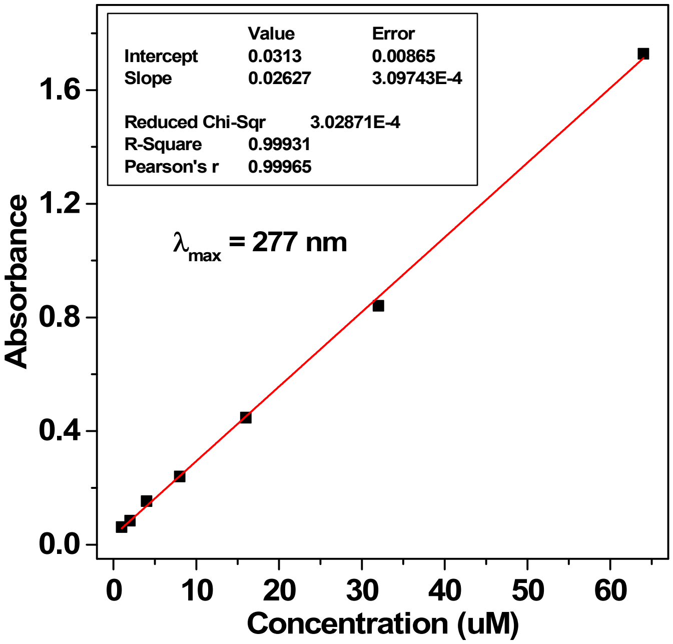

Figure S9. Plot of the absorbance of H_2QC at 277 nm as a function of concentration.

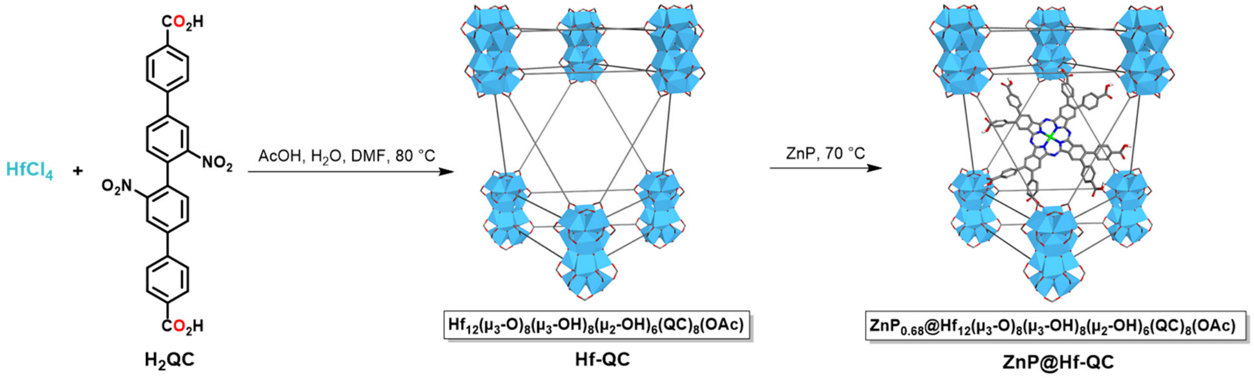

Figure S10. Synthesis of Hf-QC and ZnP@Hf-QC.

Synthesis of Hf-QC. Hf-QC was synthesized as shown in Figure S8. To a 4 mL glass vial was added 0.5 mL of $HfCl_4$ solution [2.0 mg/mL in *N,N*-dimethylformamide (DMF)], 0.5 mL of H_2QC solution (3.0 mg/mL in DMF), 75 μL of acetic acid (AcOH) and 5 μL of water. The reaction mixture was kept in an 80 °C oven for 24 hours. The off-white precipitate was collected by centrifugation and washed with DMF and ethanol.

Digestion of Hf-QC. 1.0 mg Hf-QC was dried under vacuum. To the resultant solid was added a solution of 500 μL DMSO-d_6 and 50 μL D_3PO_4. The mixture was then sonicated for 10 min, followed by the addition of 50 μL D_2O for ^{1}H NMR analysis.

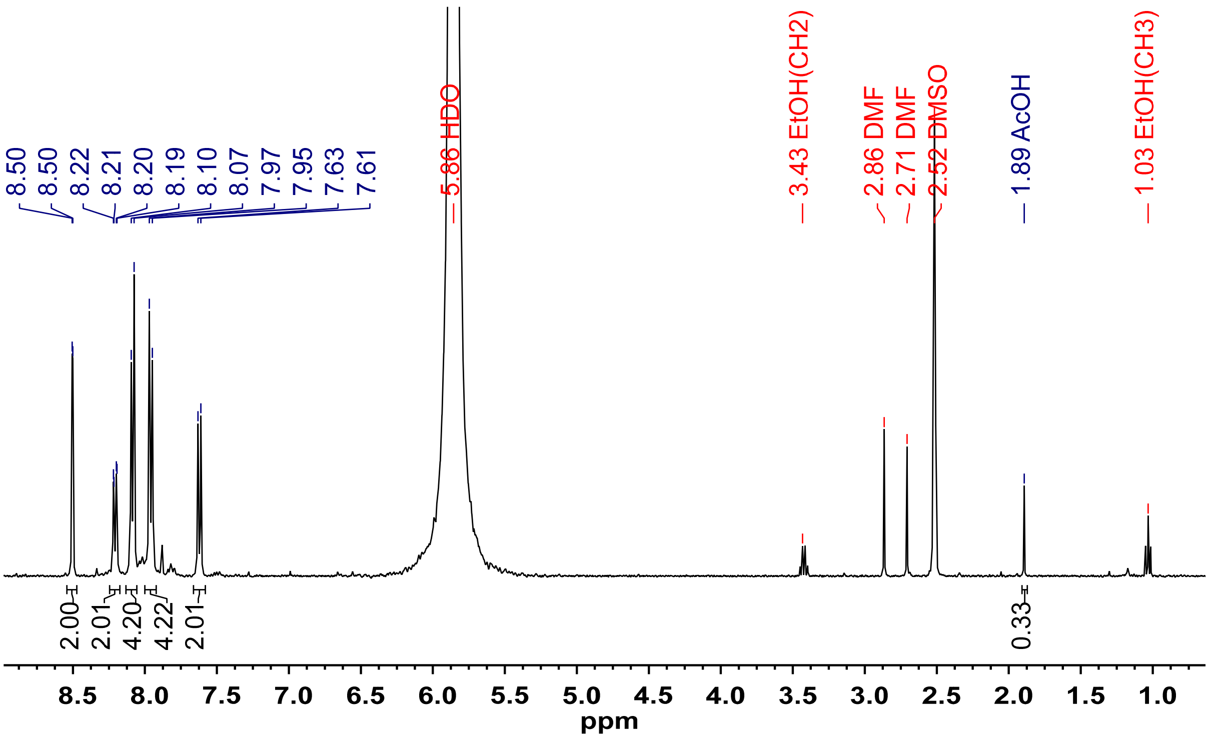

Figure S11. ^{1}H NMR spectrum of digested Hf-QC. Analysis of the integrals for the QC linker aromatic peaks (7.6-8.5 ppm) and the OAc modulator peak (1.9 ppm) gives an OAc modulator to QC linker ratio of 0.11:1, consistent with the formula $Hf_{12}(\mu_3\text{-}O)_8(\mu_3\text{-}OH)_8(\mu_2\text{-}OH)_6(QC)_{8.5}(OAc)$.

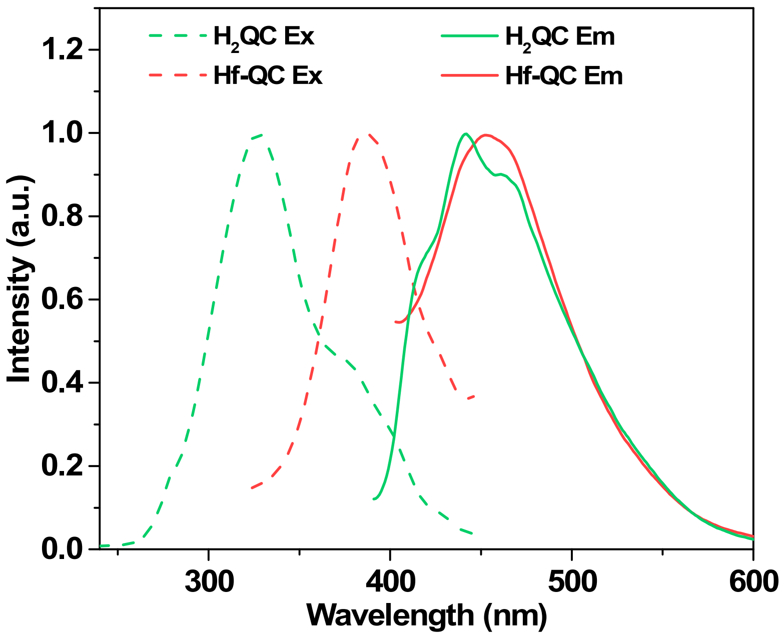

Figure S12. Normalized excitation (Ex) and emission (Em) spectra of Hf-QC and H_2QC in DMSO.

Synthesis of ZnP@Hf-QC. To a 4 mL glass vial was added 0.5 mL of Hf-QC solution (1.53 mM in EtOH based on Hf), 0.5 mL of ZnP solution (1.0 mg/mL in DMF) and a stir bar. The mixture was stirred at 70 °C overnight in an oil bath, collected by centrifugation and washed twice with DMF and dispersed in EtOH.

The weight% loading of ZnP in ZnP@Hf-QC was calculated to be 13.6% based on UV-Vis spectroscopy, which gave a ZnP concentration of 69.5 μM from the characteristic absorption peak at 700 nm, and ICP-MS, which gave a Hf concentration of 1.23 mM. The loading of ZnP@Hf-QC was calculated using the formula $(ZnP)_{0.68}@Hf_{12}(\mu_3\text{-}O)8(\mu_3\text{-}OH)_8(\mu_2\text{-}OH)_6(QC)_{8.5}(OAc)$, which was determined based on UV-Vis and ^1H NMR analysis of digested ZnP@Hf-QC and confirmed by TGA.

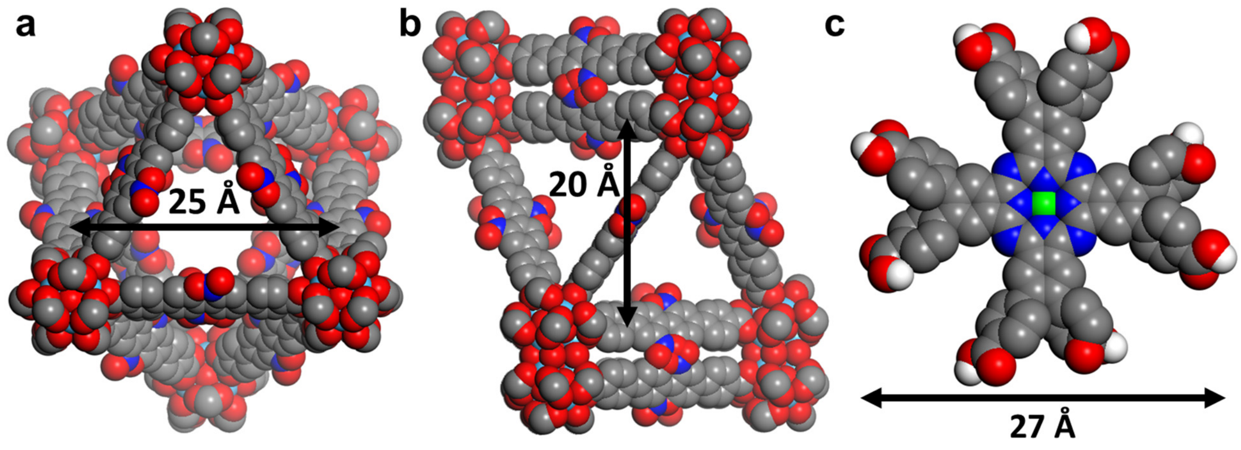

Figure S13. Space-filling model of the octahedral cavity of **pristine** Hf-QC viewed along the (a) *c*-axis and (b) *a*-axis. (c) Space-filling model of ZnP. Because of the defect and the non-closed cage structure of MOF pores, guest molecules larger than the pore diameter can still diffuse through the MOF (particularly at elevated temperatures) and are tightly confined in the pores.

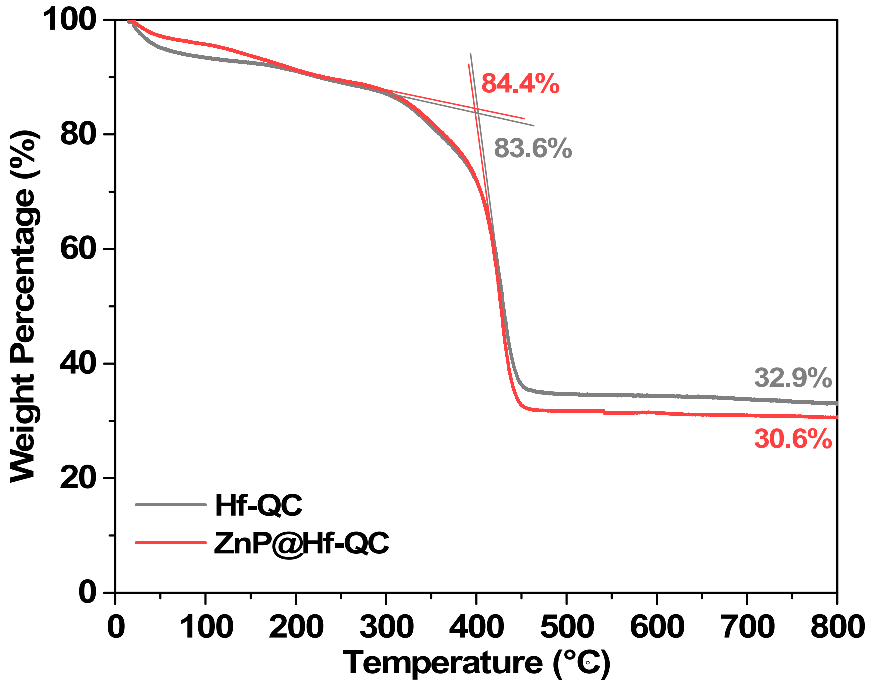

Figure S14. TGA analysis of Hf-QC and ZnP@Hf-QC. The weight loss of Hf-QC (32.9% / 83.6% = 39.4%) in the 300-800 °C range corresponds to decomposition of $Hf_{12}(\mu_3\text{-}O)_8(\mu_3\text{-}OH)_8(\mu_2\text{-}OH)_6(QC)_{8.5}(OAc)$ to $12HfO_2$, which has an expected weight loss of 37.9%. The weight loss of ZnP@Hf-QC (30.6% / 84.4% = 36.3%) in the 300-800 °C range corresponds to decomposition of $ZnP_{0.68}@Hf_{12}(\mu_3\text{-}O)_8(\mu_3\text{-}OH)_8(\mu_2\text{-}OH)_6(QC)_{8.5}(OAc)$ to $(12HfO_2 + 0.68ZnO)$, which has an expected weight loss of 34.1%.

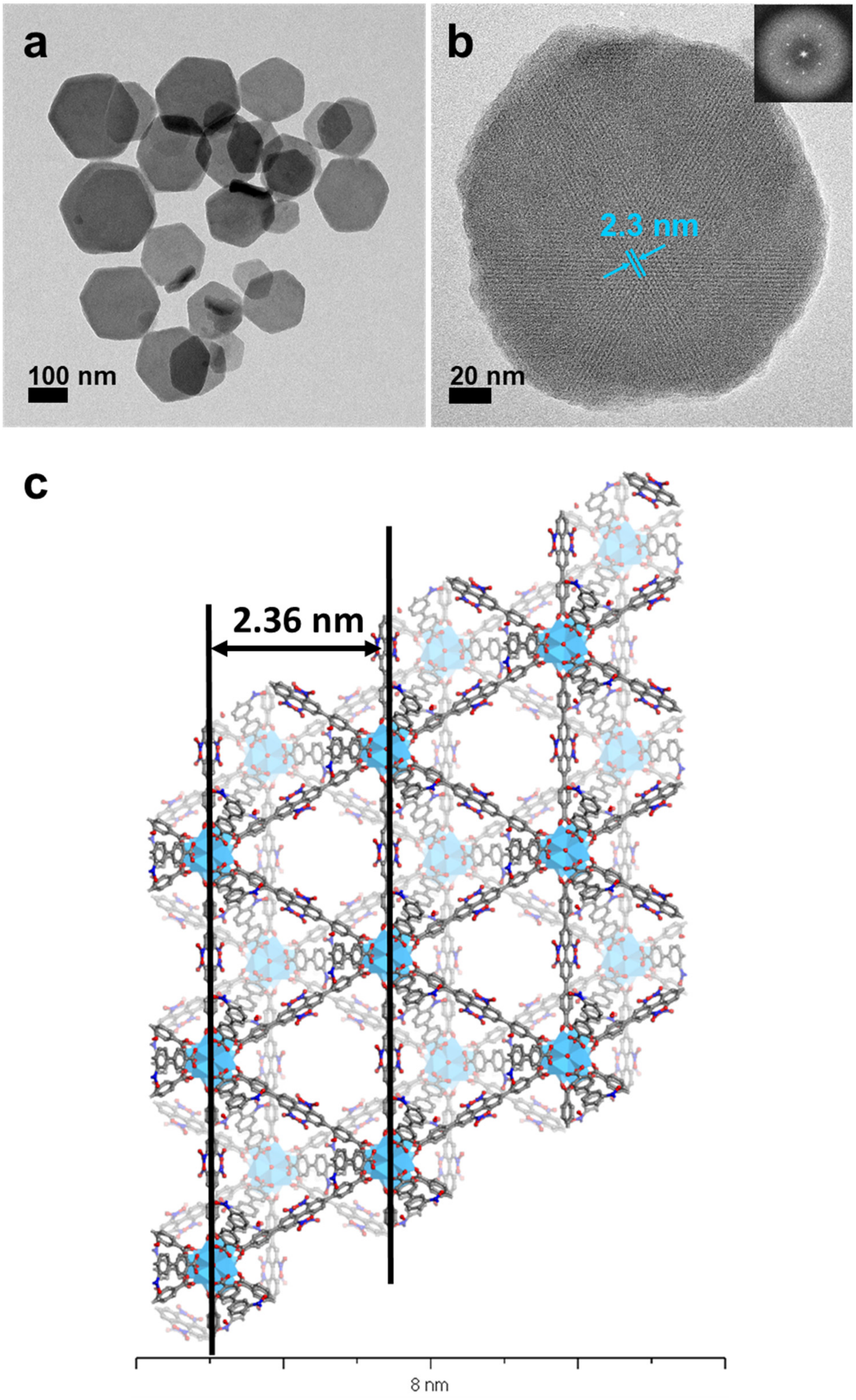

Figure 15. (a) Large-area TEM image and (b) HRTEM image with its FFT (inset) of ZnP@Hf-QC. (c) Distances in the modelled structure of Hf-QC corresponding to the lattice spacings in the HRTEM images.

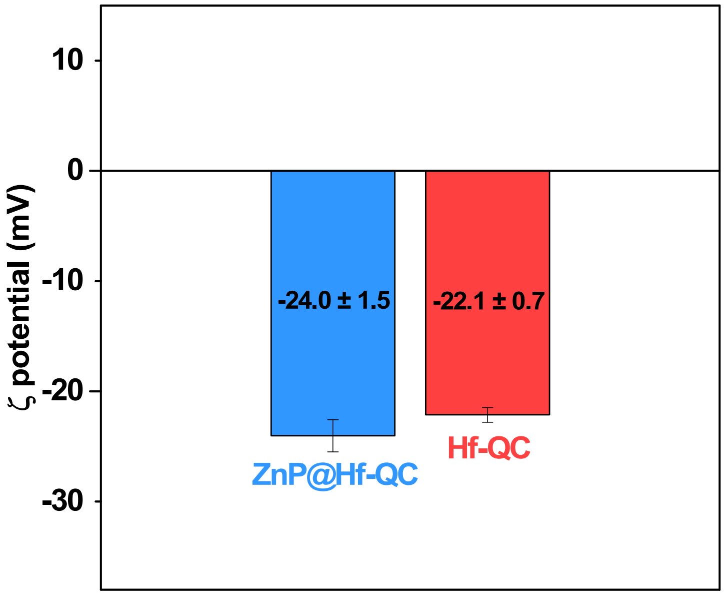

Figure S16. Zeta potentials of Hf-QC and ZnP@Hf-QC in water.

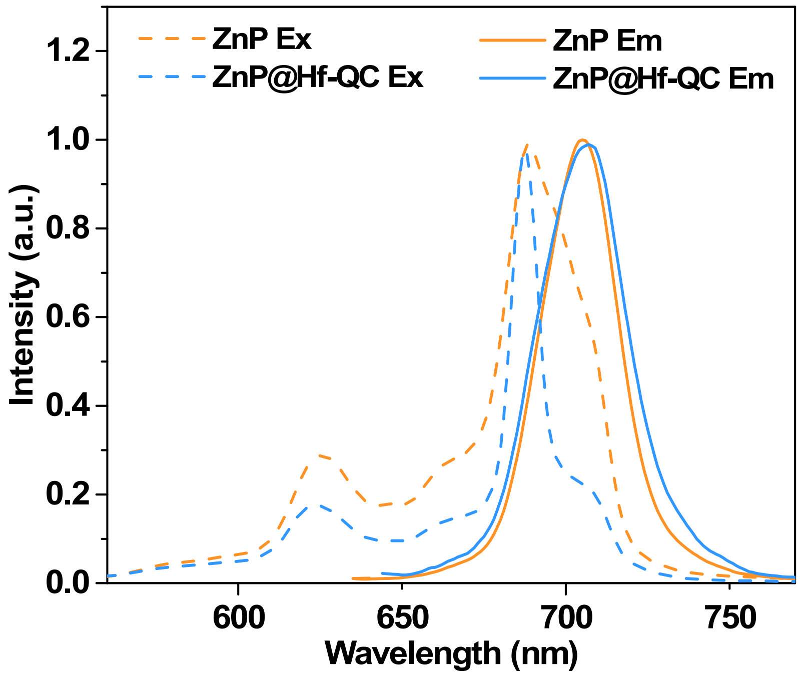

Figure S17. Normalized excitation (Ex) and emission (Em) spectra of ZnP@Hf-QC and ZnP in DMSO.

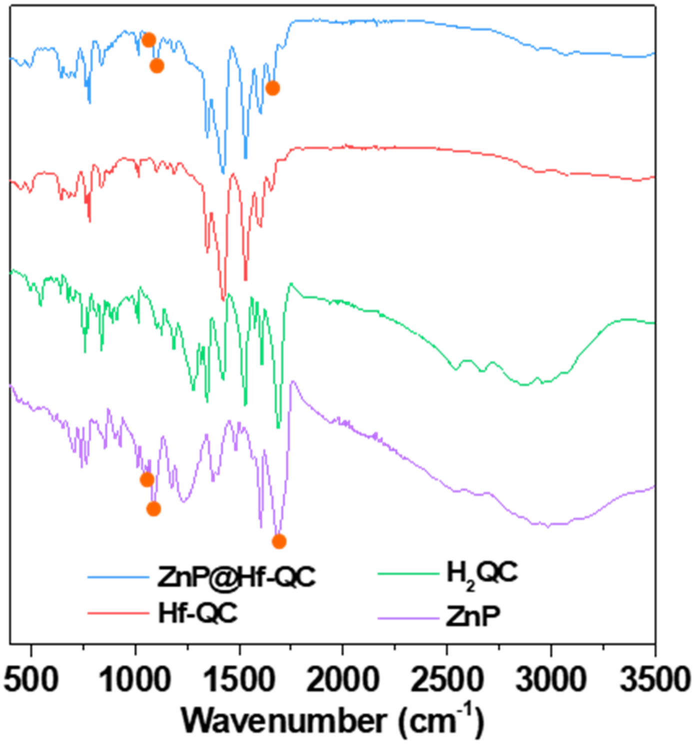

Figure S18. IR spectra of ZnP@Hf-QC, Hf-QC, H_2QC and ZnP. The characteristic IR peaks for ZnP are highlighted with orange circles.

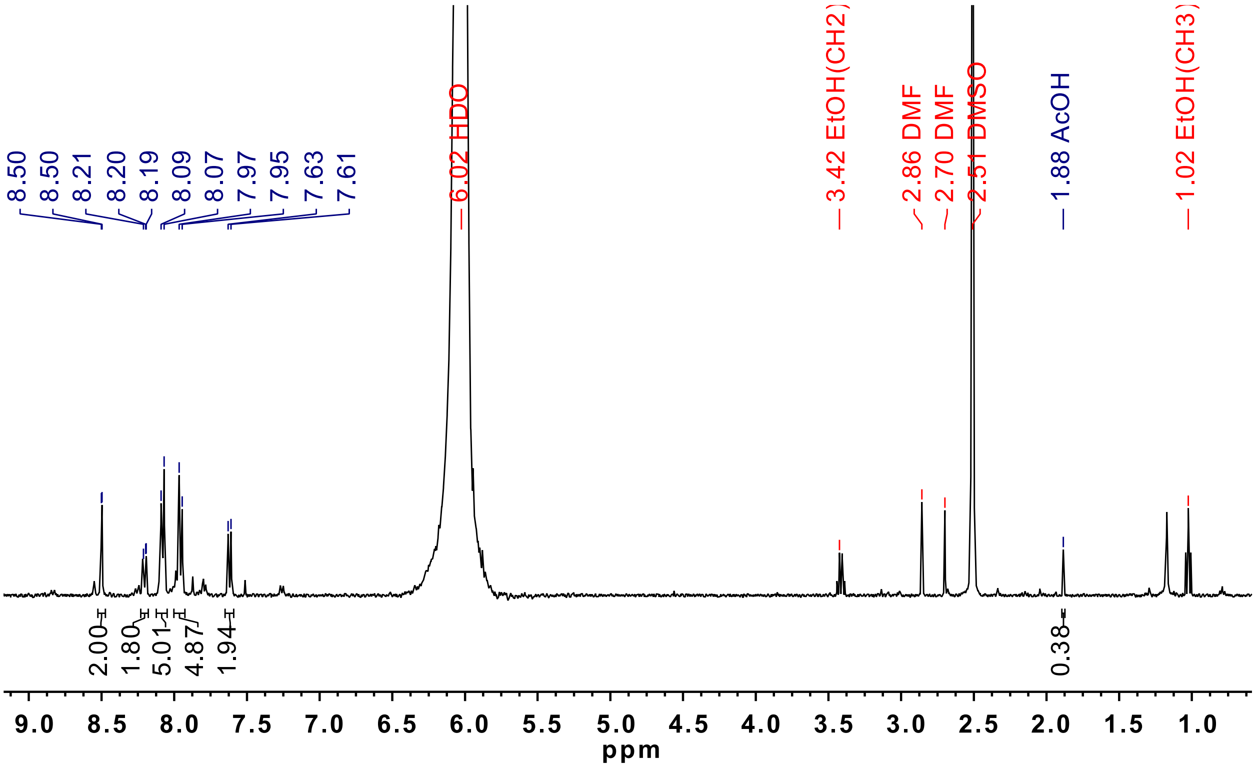

Figure S19. ^1H NMR spectrum of digested ZnP@Hf-QC. Digested ZnP@Hf-QC maintains the same OAc modulator to QC linker ratio (0.11:1) as Hf-QC, consistent with the formula $(ZnP)_{0.68}@Hf_{12}(\mu_3\text{-}O)_8(\mu_3\text{-}OH)_8(\mu_2\text{-}OH)_6(QC)_{8.5}(OAc)$ and confirming the physical loading of ZnP.

S3. Reactive Oxygen Species Generation

The time-dependent $^1O_2$ generation of ZnP, H_2QC, Hf-QC and ZnP@Hf-QC was detected by singlet oxygen sensor green (SOSG, Life Technologies, USA) assay upon light irradiation. ZnP, H_2QC, Hf-QC and ZnP@Hf-QC suspensions were prepared with an equivalent dose of 0.5 μM ZnP and 71.8 μM QC in PBS. To 2 mL of each of these suspensions, an SOSG stock solution (5 μL at 5 mM) was added (final SOSG concentration = 12.5 μM) before fluorescence measurements. The mixed solution was exposed to an LED light (700 nm, 100 mW/cm^2) for 0, 10, 20, 30, 60, 120, 180, 300, 420 and 600 seconds and the fluorescence intensity at different time points was measured by a fluorimeter.

S4. *In vitro* study

S4.1. Cellular uptake

The cellular uptake of ZnP, Hf-QC and ZnP@Hf-QC was evaluated on CT26 cells. The cells were seeded on 6-well plates at a density of 5×10^5/well followed by overnight culture. First, ZnP and ZnP@Hf-QC were added to each well to reach an equivalent ZnP concentration of 5 μM in medium (N=3). The cells were incubated at 37°C for 1, 2, 4, and 8 hours. At each time point, the medium was aspirated, the cells were washed with DPBS for three times, trypsinized and collected by centrifugation and counted by a hemocytometer. The cell pellets were digested with a mixture of DMSO and 10% H_3PO_4 in 1.5 mL Eppendorf tubes for 48 hours with strong vortex and sonication every 12 hours and the ZnP concentration was determined by UV-Vis absorbance at 700 nm (ε=422,000 $M^{-1}\cdot cm^{-1}$). Then the uptake of ZnP@Hf-QC and Hf-QC was measured in the same way at an equivalent Hf concentration of 85.7 μM except the digestion step where] the cell pellets were treated with 99% of concentrated HNO_3 (67-70% trace metal grade) and 1% of hydrofluoric acid in 1.5 ml ep tubes for 48 hours with strong vortex and sonication every 12 hours. The Hf concentration was then determined by ICP-MS.

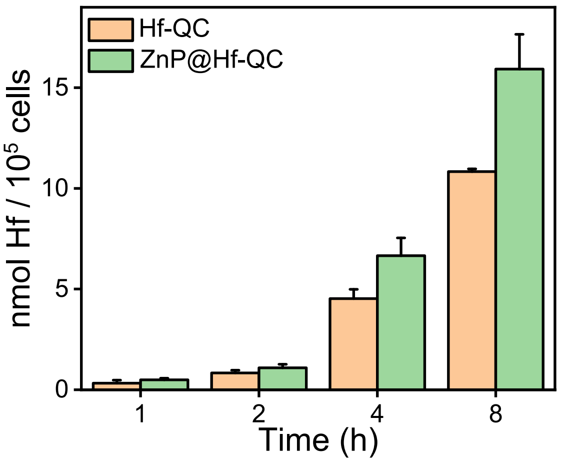

Figure S20. Time-dependent cellular uptake of Hf-QC and ZnP@Hf-QC (N=3)

In addition, the relative cellular uptake was also confirmed by flow cytometry and confocal microscopy after 8-hour incubation of CT26 cells with ZnP, Hf-QC and ZnP@Hf-QC. The fluorescence intensity of ZnP was monitored by APCR700 channel (ex. 640 nm, em. 730/45 nm).

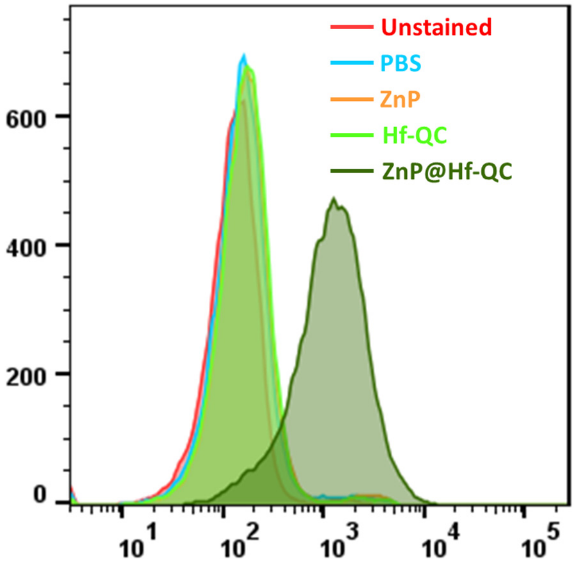

Figure S21. Cellular uptake of ZnP, Hf-QC and ZnP@Hf-QC by flow cytometry (quantified by ZnP's fluorescence in APCR700 channel).

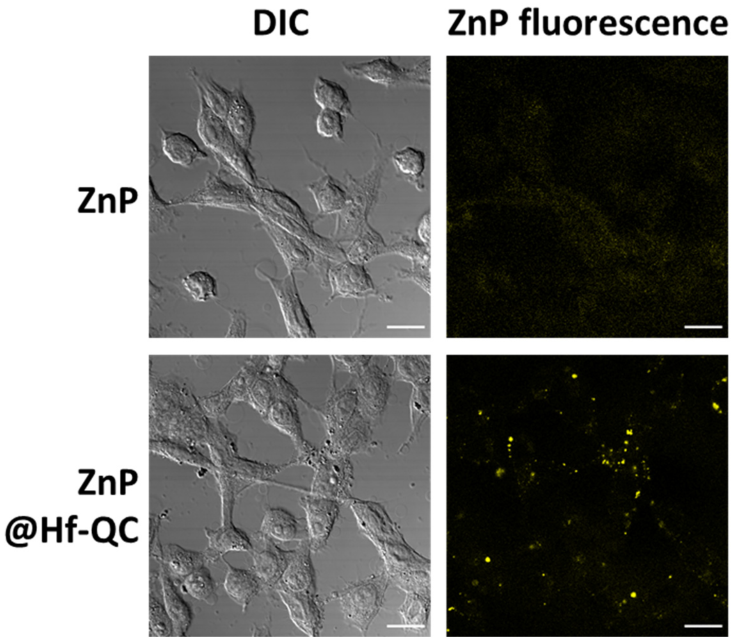

Figure S22. Fluorescence signals of ZnP (yellow) in CT26 cells observed by CLSM (scale bars = 20 μm).

The colocalization between ZnP (red channel) and LysoTracker (green channel) was not only verified by ROI analysis in Figure 4d, 4e & 4f, but was also confirmed by whole image analysis. The 2D intensity profiles and Pearson's coefficient were calculated by Coloc2 plugin in ImageJ software. From 0.5 h to 24 h, the R value was increasing and ZnP had more signals overlapping with LysoTracker.

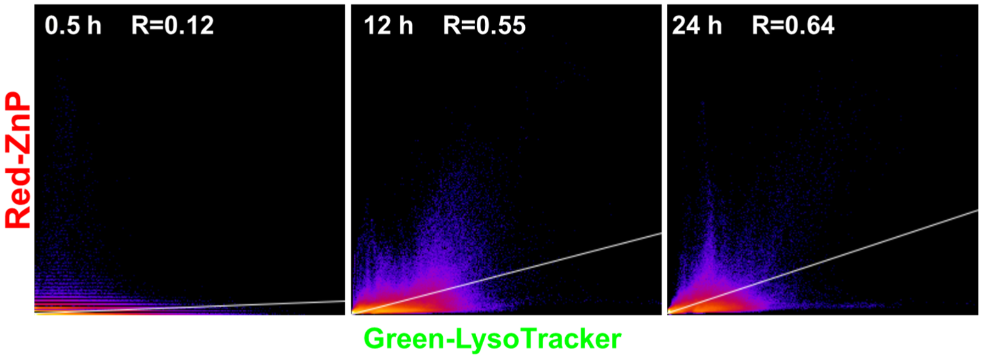

Figure S23. 2D intensity profiles of colocalization between ZnP (red channel) and LysoTracker (green channel) by Coloc2 plugin in ImageJ software.

S4.2. *In vitro* cytotoxicity

Cell viability assay. The cytotoxicity of ZnP, Hf-QC and ZnP@Hf-QC was evaluated on CT26 cells by the MTS assay. The cells were first seeded on 96-well plates at a density of 10000 cells/mL with 100 μL RPMI medium per well and further cultured overnight. ZnP or ZnP@Hf-QC was added to the wells at an equivalent ZnP concentration of 0, 20, 50, 100, 200, 500, 1000 and 2000 nM and incubated for 8 hours (N=6), followed by light irradiation (700 nm, 100 mW/cm^2, 10 min, 60 J/cm^2 as total dose). Hf-QC was added to the wells at an equivalent Hf concentration of 0, 0.34, 0.86, 1.7, 3.4, 8.6, 17.1, 34.3 μM, followed by the same PDT treatment above. During light irradiation, a water jacket was placed above the plate to avoid heating of the plate by the LED. Then the cells were further incubated for 48 hours and the cell viability was determined by MTS assay. IC_{50} value of ZnP@Hf-QC(+) was determined as 139 nM by fitting the dose response curves in Origin Lab. No significant toxicity of ZnP(+) or Hf-QC(+) was found until 2 μM. No obvious dark toxicity was observed for ZnP(-), Hf-QC(-) and ZnP@Hf-QC(-).

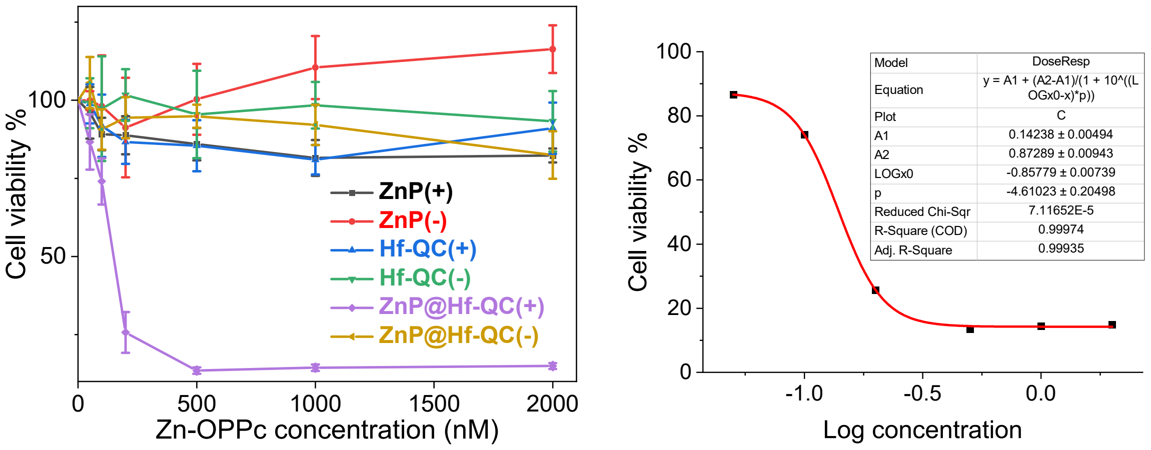

Figure S24. Cell viability curves (left) and IC_{50} calculation (right) of ZnP and ZnP@Hf-QC with (+) and without (-) light irradiation on CT26 cells (N=6).

Live cell imaging. To further verify the PDT killing effect by ZnP@Hf-QC(+), we observed cell proliferation and morphology of CT26 cells after PDT treatment by IncuCyte S3. CT26 cells were first seeded in 96-well plate with a density of 1500 cells / well and cultured overnight. Then ZnP@Hf-QC was added at an equivalent ZnP concentration of 1 μM and further incubated for 8 hours, followed by light irradiation (700 nm, 100 mW/cm^2, 10 min, 60 J/cm^2 as total dose). Then the plate was put in the IncuCyte S3 for 46 hours. The phase contrast images were acquired and the movies of cell proliferation were shown in movies S1 and S2 in the supporting information.

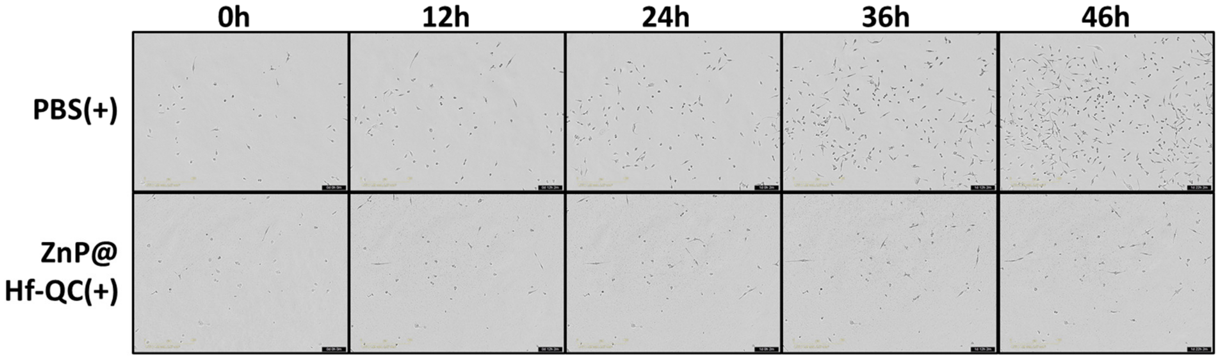

Figure S25. Live cell images of PDT treated cells by IncuCyte S3.

Apoptotic cell death. The apoptosis after PDT treatment was evaluated on CT26 cells by flow cytometry and confocal laser scanning microscopy (CLSM). For flow cytometry analysis, on two 6-well plates, CT26 cells were seeded at a density of $2.5x10^5$ cells/ml with RPMI medium and cultured overnight. The cells on both plates were treated with ZnP, Hf-QC, or ZnP@Hf-QC at an equivalent ZnP concentration of 2 μM (or Hf concentration of 34.3 μM) and further incubated for 8 hours. Then one of the plates was irradiated with LED light (700 nm, 100 $mW \cdot cm^{-2}$) for 10 min. The cells on both plates were washed with cold DPBS, exchanged to warm fresh medium and further incubated for another 24 hours. The cells were then trypsinized and stained with Alexa Fluor 488 Annexin V/dead cell apoptosis kit (1:20 dilution in 1x binding buffer) and propidium iodide (PI, 1 μg/mL, 15 min on ice) (Thermo Fisher Scientific, USA) following vendor's protocol for flow cytometry analysis. For CLSM analysis, inside 35 mm glass bottom dishes, CT26 cells were seeded at a density of $1x10^5$ cells/ml with RPMI medium and cultured overnight. Then the treatment and staining were the same with flow cytometry except the addition of a counterstain step of Hoechst-33342 3 μg/mL 5 min at RT and fixation by 2% PFA (in 1x binding buffer). The dishes were then washed by DPBS and added with 1 mL 1x binding buffer and observed under Leica Stellaris 8 microscope immediately.

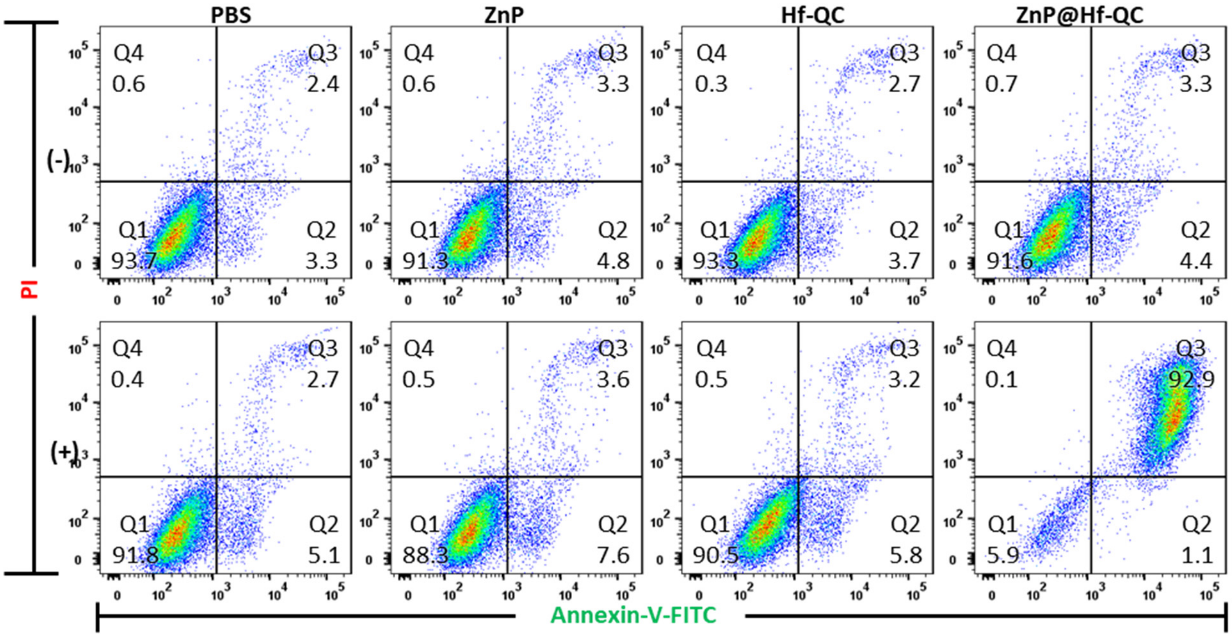

Figure S26. Flow cytometry analysis of CT26 cell apoptosis 24 hours after PDT treatment. Q1, Q2, Q3 and Q4 indicate normal, early apoptotic, late apoptotic, necrotic populations among CT26 cells, respectively. The percentages of each population were shown in each quadrant.

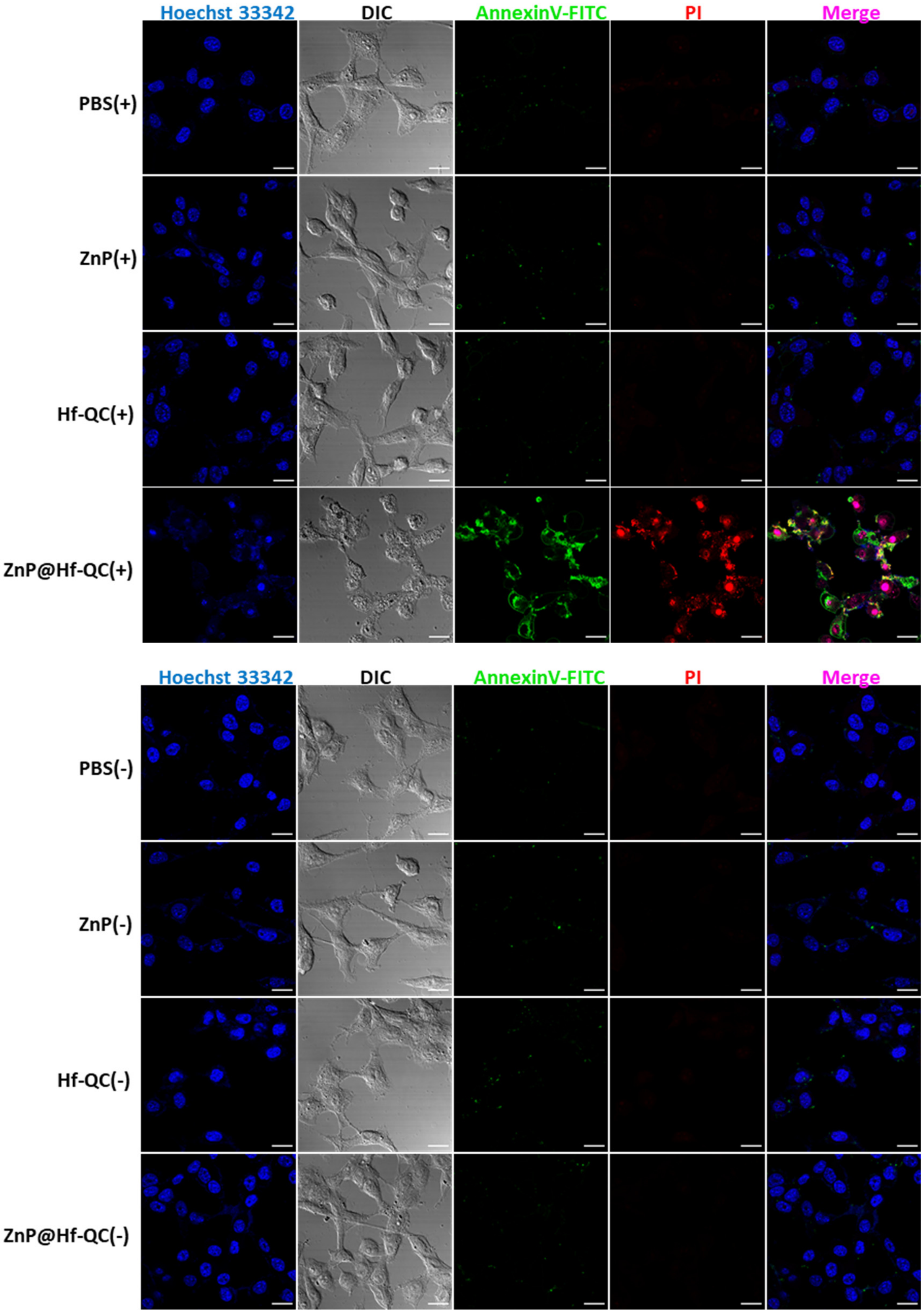

Figure S27. CLSM images of CT26 cell apoptosis 24 hours after PDT treatment (Hoechst, blue; Annexin-V-FITC, green; PI, red; Merge, pink; scale bars are 20 μm).

S4.3. *In vitro* ROS generation

The ROS generation of ZnP, Hf-QC or ZnP@Hf-QC during PDT treatment was evaluated on CT26 cells by flow cytometry and CLSM. For flow cytometry analysis, on two 6-well plates, CT26 cells were seeded at a density of $2.5x10^5$ cells/ml with RPMI medium and cultured overnight. The cells on both plates were treated with ZnP, Hf-QC, or ZnP@Hf-QC at an equivalent ZnP concentration of 2 μM (or Hf concentration of 34.3 μM) and further incubated for 7 hours. 20 μM DCF-DA (Invitrogen) was then added to each well for another 1-hour incubation. Then one of the plates was irradiated with LED light (700 nm, 100 mW·cm^{-2}) for 10 min. The cells were then washed with PBS, trypsinized, and analyzed by flow cytometry. For CLSM, inside 35 mm glass bottom dishes, CT26 cells were seeded at a density of $1x10^5$ cells/ml with 2 mL RPMI medium and cultured overnight. The cells were treated in the same way as flow cytometry but not detached. The cells were washed with DPBS three times, exchanged with cold DPBS and mounted for confocal imaging immediately using Leica Stellaris 8 microscope.

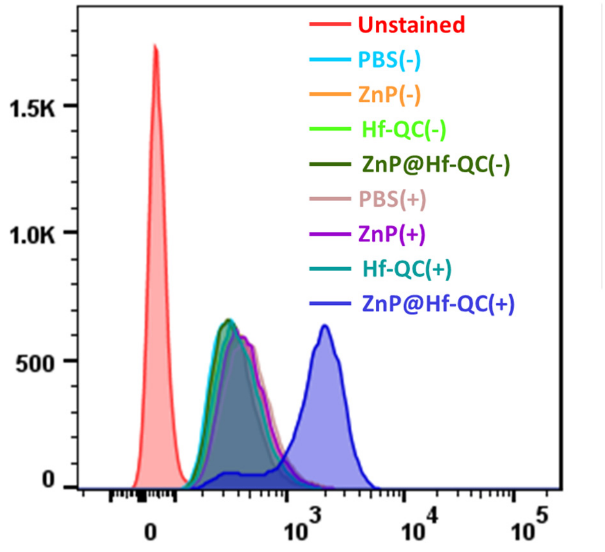

Figure S28. Histograms of intracellular ROS signals quantified by flow cytometry. The negative control without staining was shown in red (DCF-DA, FITC channel).

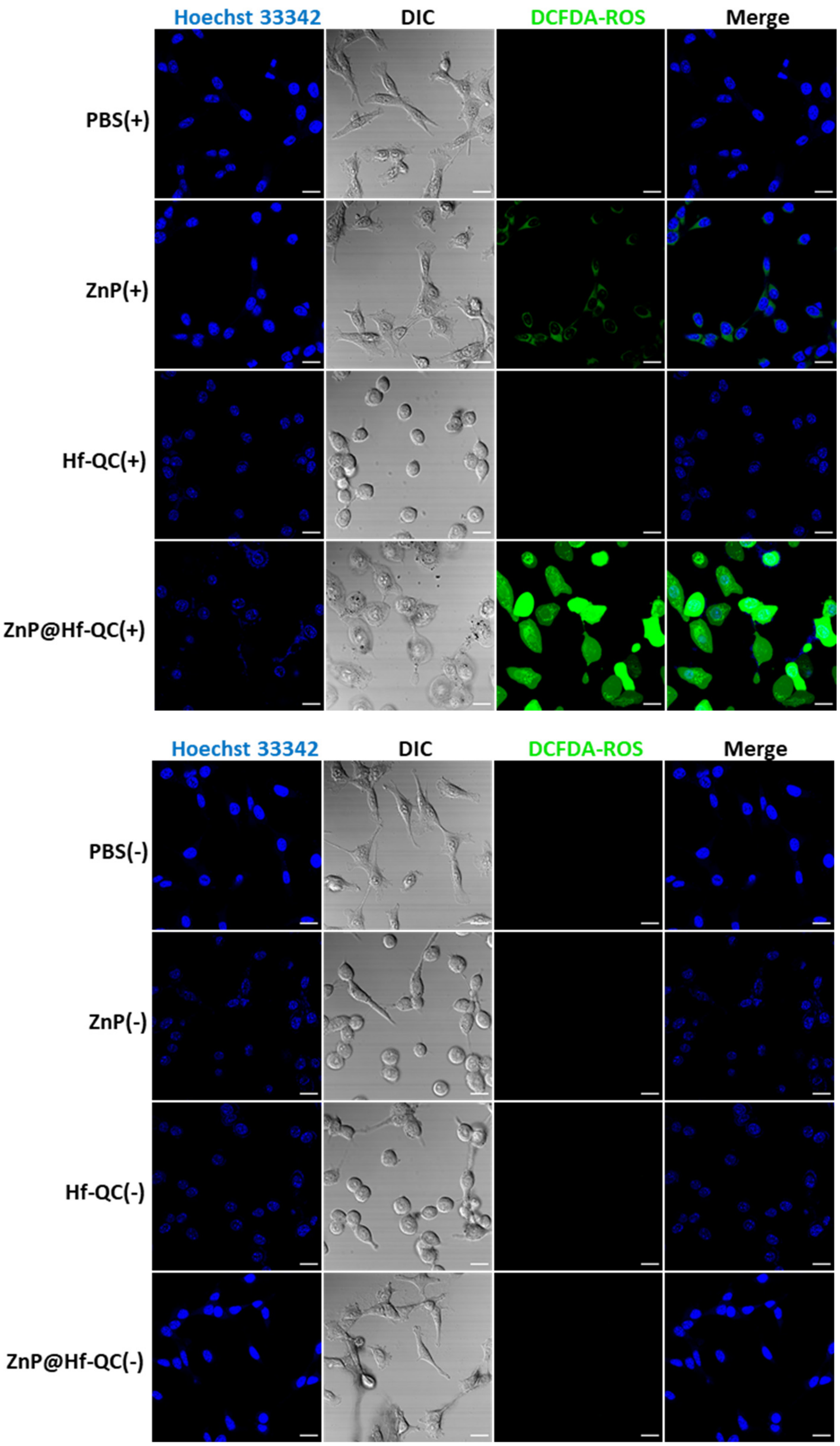

Figure S29. CLSM images of ROS generation in cells triggered by PDT (DCF-DA: green; scale bars are 20 μm).

S4.4. CRT expression

By flow cytometry and CLSM, immunogenic cell death was investigated by Calreticulin (CRT) expression level after PDT treatment. For flow cytometry analysis, CT26 cells were seeded on two 6-well plates at a density of 2.5x10^5 cells/ml with 2 mL RPMI medium and cultured overnight. The cells were treated with ZnP, Hf-QC or ZnP@Hf-QC at an equivalent ZnP concentration of 2 μM (or Hf concentration of 34.3 μM) and further incubated for 8 hours. Then one of the plates was irradiated with LED light (700 nm, 100 mW·cm^{-2}) for 10 min. The cells on both plates were washed with DPBS to remove excess ligands or Hf-QC, exchanged with warm fresh medium and further incubated for another 24 hours. The medium was then discarded, and cells were washed with DPBS and trypsinized to obtain cell suspension. The cells were stained with anti-Calreticulin Alexa Fluor 488 (NOVUS) (1:150 dilution in 0.5% BSA DPBS solution) on ice for 30 min, washed with PBS once and resuspended in FACS buffer (0.5% BSA, 2 mM EDTA and 0.05% NaN_3 in DPBS) for flow cytometry analysis. As for CLSM analysis, the PDT treatment procedure was the same with flow cytometry except MC38 cells were seeded with a coverslip in each well with a cell density diluted to 1x10^5 cells/mL. At 24 hours after treatment, the cells were fixed with -20 °C methanol for 5 min, blocked with 3% BSA and 1% FBS at RT for 1h, and then stained with anti-Calreticulin Alexa Fluor 488 (NOVUS) (1:150 dilution in 0.5% BSA DPBS solution) at 4 °C overnight. The cells were washed with DPBS and counterstained with Hoechst (3 μg/mL 5 min RT), and the coverslips were mounted on glass slides with ProLong™ Glass Antifade Mountant, cured for 6 hours, and sealed for confocal imaging under Leica Stellaris 8 microscope.

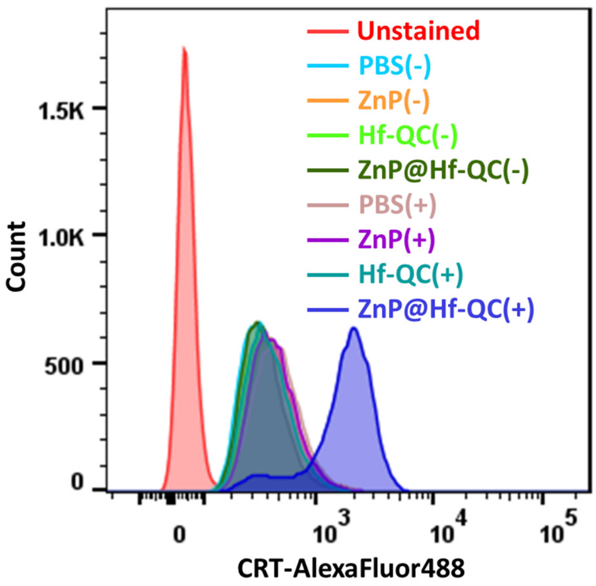

Figure S30. Flow cytometry analysis of CRT expression on CT26 cells 24 hours after PDT treatment. The negative control without staining was shown in red (CRT, FITC channel).

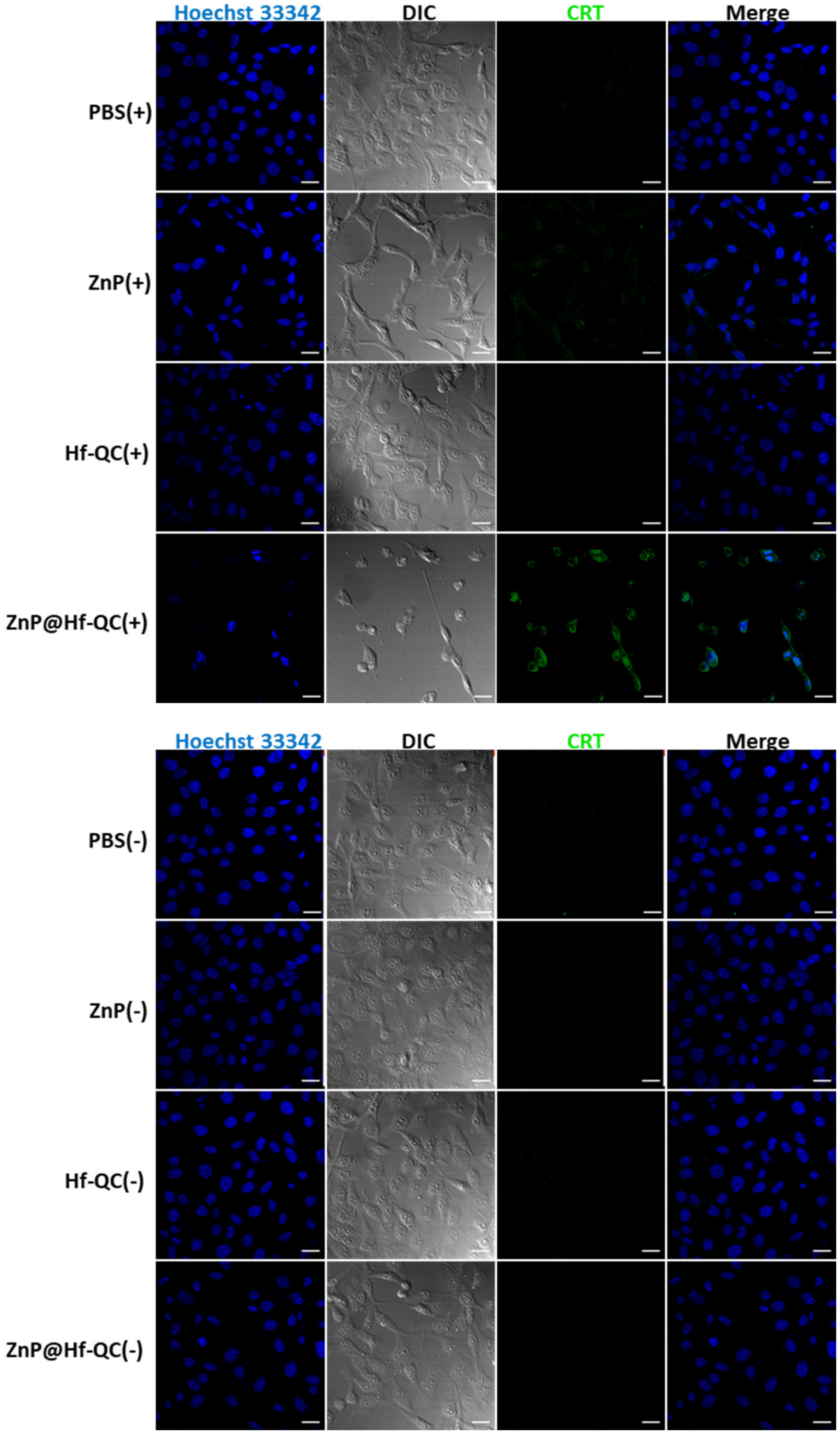

Figure S31. CLSM images of CRT expression on the surfaces of MC38 cells induced by PDT 24 hours later (DNA, Hoechst; CRT, green; scale bars are 20 μm).

S5. *In vivo* study

S5.1. *In vivo* anti-cancer efficacy

Hf-QC and ZnP@Hf-QC were PEGylated before intravenous administration. Briefly, the nMOF was first dispersed in water and the same weight amount of DSPE-PEG(2000) was added. The mixture was stirred at r.t. for 4 hours and lyophilized to afford a solid. The solid was suspended in PBS immediately before use. To evaluate *in vivo* PDT efficacy of ZnP@Hf-QC, CT26 tumor-bearing BALB/c and MC38 tumor-bearing C57Bl/6 mouse models were established by inoculating 2x10^6 cells/mouse subcutaneously at day 1. At day 7, 25 mice with tumor volume around 100 mm^3 were randomized for PDT treatment. ZnP, Hf-QC or ZnP@Hf-QC was injected intravenously with equivalent ZnP dose of 50 nmol (or Hf dose of 0.88 μmol) (N=5). Control group was treated with PBS (N=5). After 12 hours, the mice were anaesthetized with 2.5% (V/V) isoflurane/O_2 and only the tumor area was irradiated with LED light (700 nm, 100 mW/cm^2, 10 min). Tumor sizes were measured by an electronic caliper (tumor volume = length×width^2/2) and body weight was monitored. At day 22 and day 24 for CT26 and MC38 model, respectively, the mice were sacrificed, and the tumors were weighed, photographed and sectioned for H&E and TUNEL staining. Major organs were sectioned for hematoxylin-eosin (H&E) staining to evaluate biosafety. The tumor growth inhibition index (TGI) was defined as the equation below:

$$TGI = 1 - \frac{\frac{T_e}{T_s} \Big/ \frac{C_e}{C_s}}{1 - \frac{C_s}{C_e}} \times 100\%$$

where T_e, T_s, C_e, and C_s represent average tumor volumes of treated mice at endpoint, treated mice at starting-point, control mice at endpoint and control mice at starting-point, respectively.

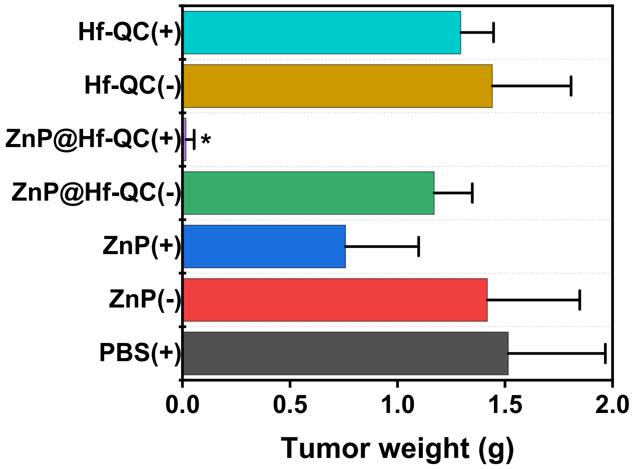

Figure S32. Weight of excised CT26 tumors of BALB/c mice in all groups.

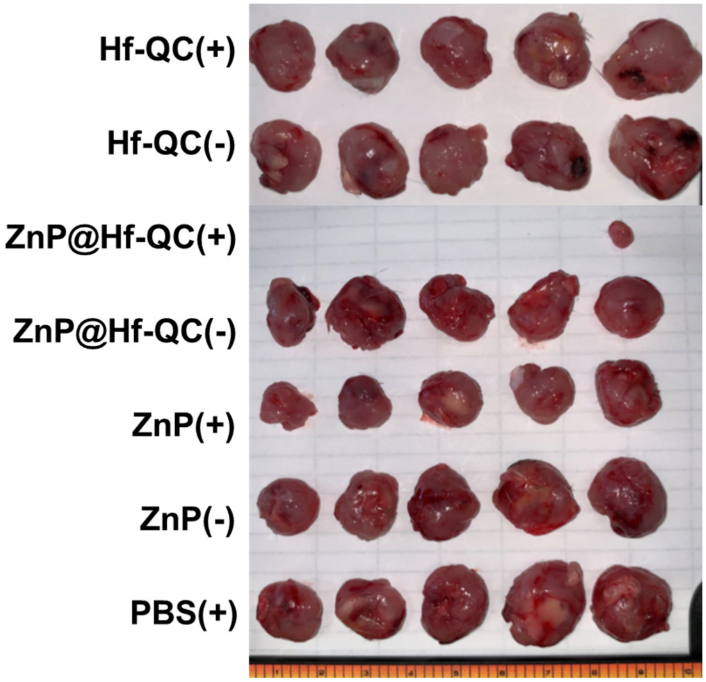

Figure S33. Photograph of excised CT26 tumors of BALB/c mice in all groups.

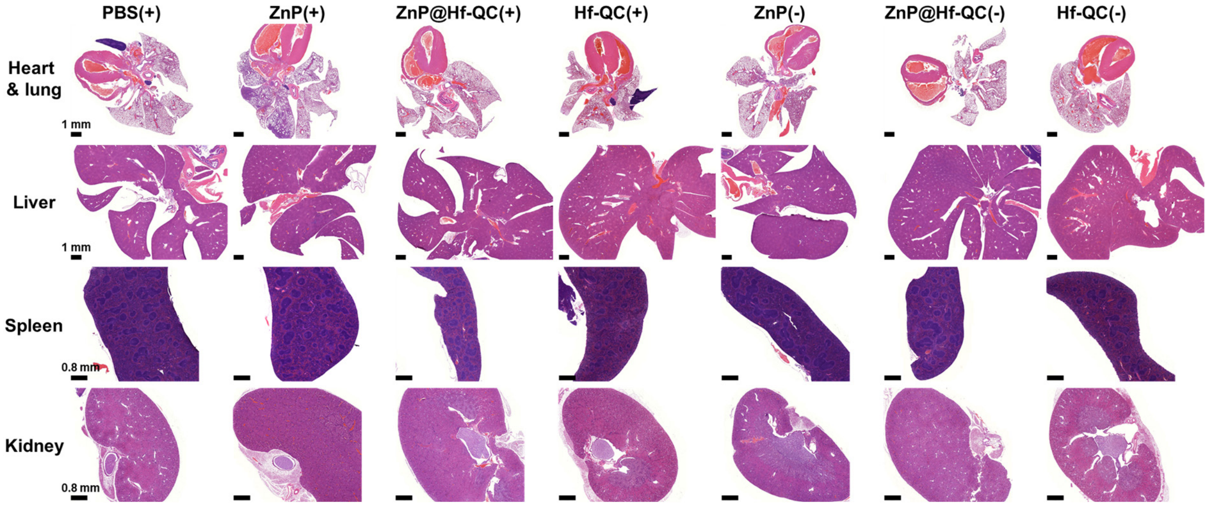

Figure S34. H&E staining of major organs from CT26 tumor-bearing BALB/c mice in different treatment groups (scale bars for heart&lung and liver are 1 mm and scale bars for spleen and kidney are 0.8 mm).

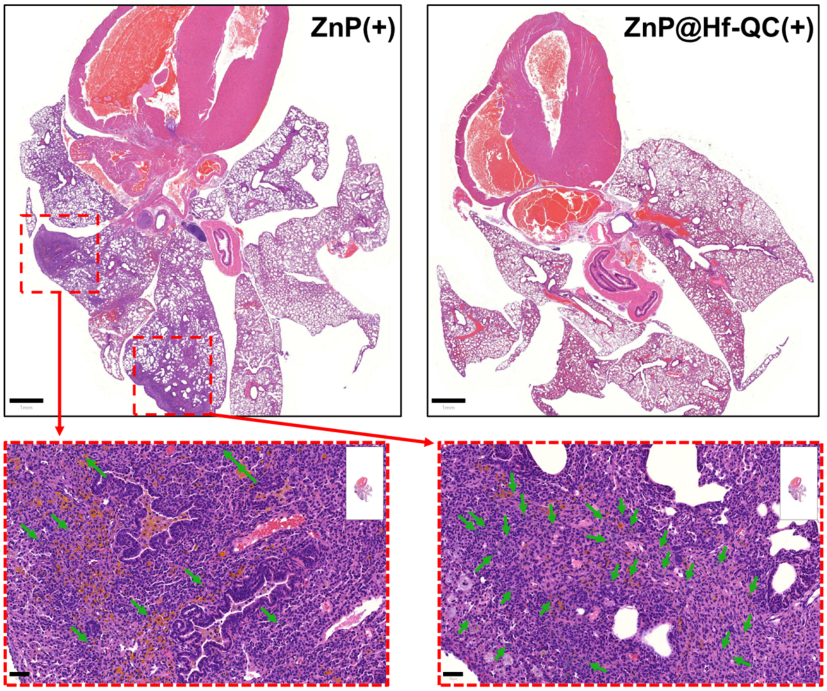

Figure S35. H&E staining showed pulmonary edema (marked by red dashed square and zoom-in views below) in ZnP treated BALB/c mice, indicating ZnP alone might cause blockade and inflammation in lungs. Green arrow showed the appearance of green objects which was ZnP. In comparison, ZnP@Hf-QC showed a healthy lung structure with no sign of particle accumulation. Scale bar = 1 mm for top figures and = 50 μm for bottom figures.

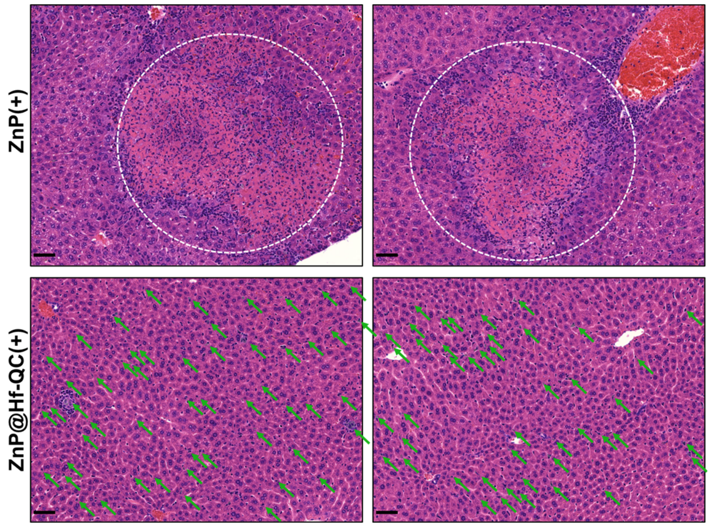

Figure S36. H&E staining showed accumulation of ZnP and ZnP@Hf-QC in liver. ZnP caused local inflammation (marked by white dashed circles) in liver and severe vacuolation of hepatocytes. However,

ZnP@Hf-QC showed a healthy liver morphology though large amounts of particles can be observed (marked by green arrows). Scale bar = 50 μm.

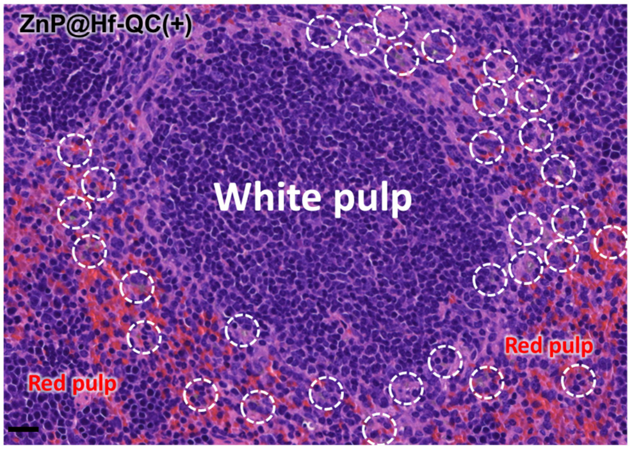

Figure S37. H&E staining showed accumulation of ZnP@Hf-QC in spleen. ZnP was barely found in spleen. ZnP@Hf-QC particles were marked by white dashed circles. No obvious abnormalities were found in the spleens of ZnP@Hf-QC treated mice. (Scale bar = 20 μm)

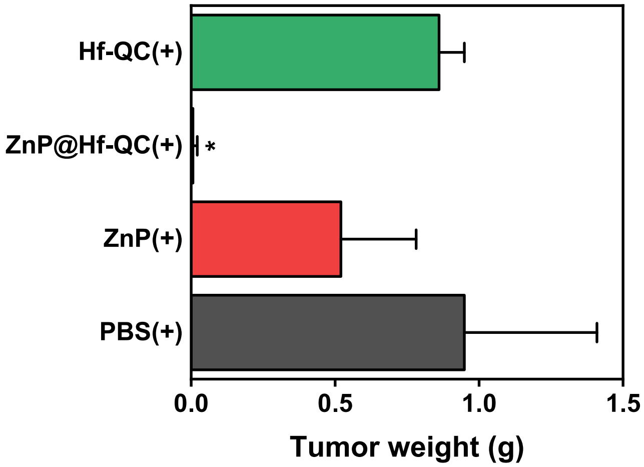

Figure S38. Weight of excised tumors of MC38 tumor-bearing C57BL/6 mice in treatment groups

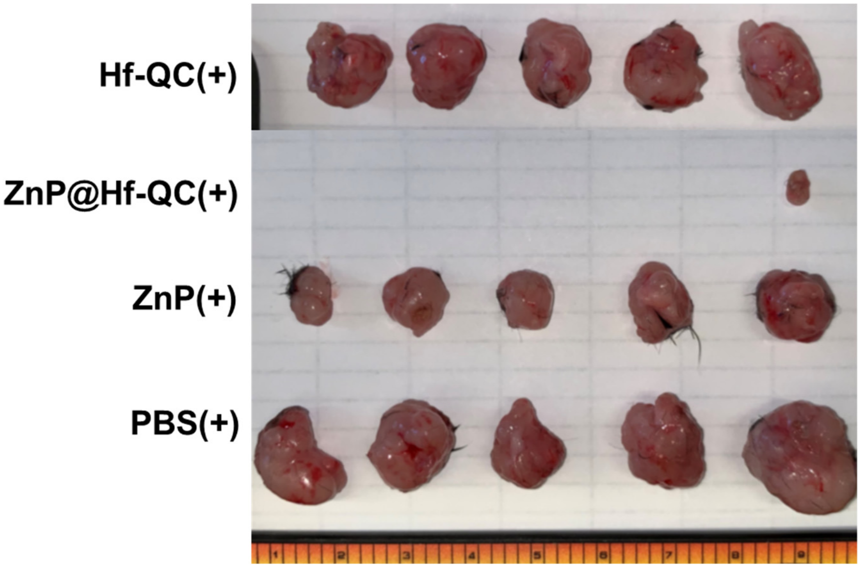

Figure S39. Photograph of excised MC38 tumors of C57BL/6 mice in treatment groups.

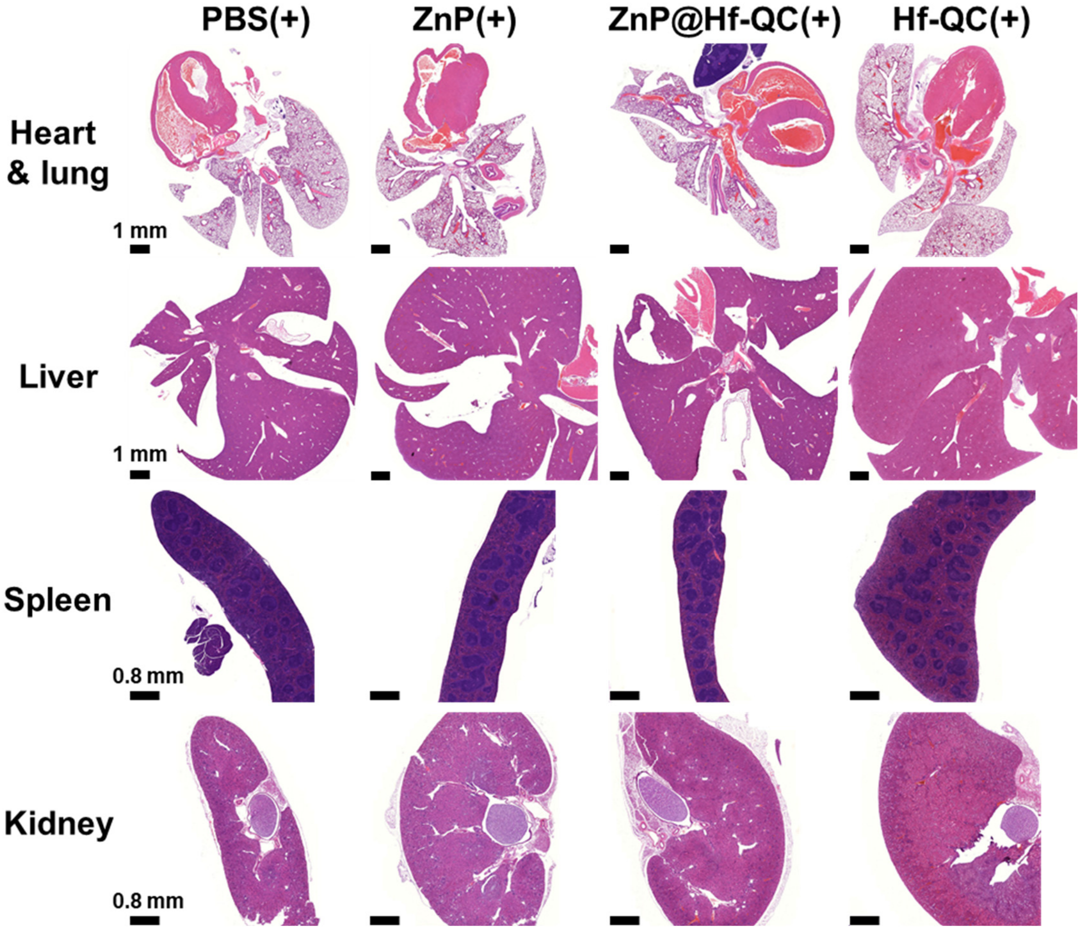

Figure S40. H&E staining of major organs from MC38 tumor-bearing C57BL/6 mice in different treatment groups (scale bars for heart&lung and liver are 1 mm and scale bars for spleen and kidney are 800 μm).

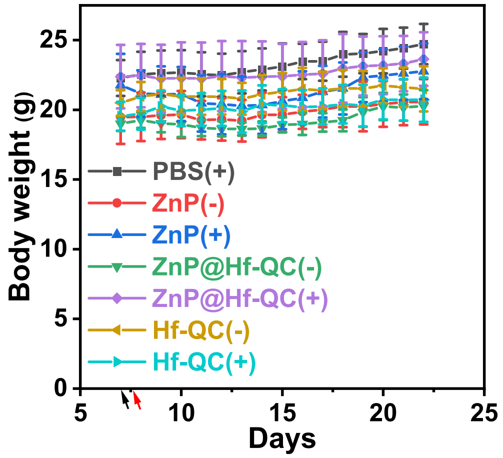

Figure S41. Body weight plot of treated CT26 bearing BALB/c mice (The black arrow refers to *i.v.* injection of different treatments and the red arrow refers to PDT treatment).

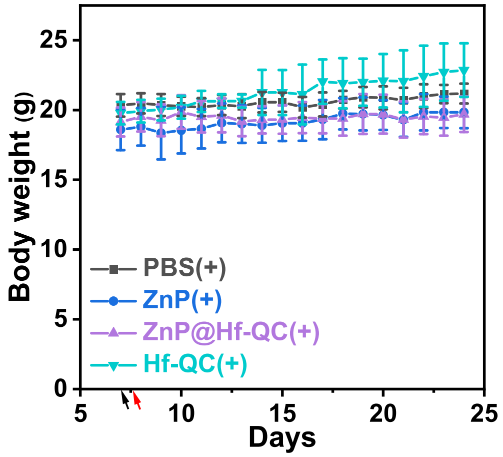

Figure S42. Body weight plot of treated MC38 bearing C57BL/6 mice (The black arrow refers to *i.v.* injection of different treatments and the red arrow refers to PDT treatment).

S5.2. *In vivo* immunogenic cell death

To evaluate *in vivo* immunogenic cell death after PDT treatment, tumors were fixed in 10% neutral buffer (Fisher Chemical) for 3 days and 70% ethanol for 1 day. Tissues were embedded in paraffin, sectioned and stained for TUNEL assay by Human Tissue Resource Center in the University of Chicago. Briefly, after deparaffinization and rehydration, tissue sections were treated with 20 μg/ml of proteinase K (S3004, DAKO) for 15 minutes. 3% hydrogen peroxidase was used to block the endogenous enzyme activity followed by equilibration buffer incubation. Working strength TDT enzyme was applied on tissue sections for 1-hour incubation at 37°C in a wet chamber. Following stop/wash Buffer wash, TdT labeled DNA fragments was visualized through anti-Digoxigenin Conjugate and DAB+ chromogen (DAKO, K3468). Tissue sections were briefly immersed in hematoxylin for counterstaining and were covered with cover glasses. The slides were then sealed and scanned on a CRi Pannoramic SCAN 40x whole slide scanner by Integrated Light Microscopy Core in the University of Chicago. The images were analyzed by QuPath-0.2.3 software.

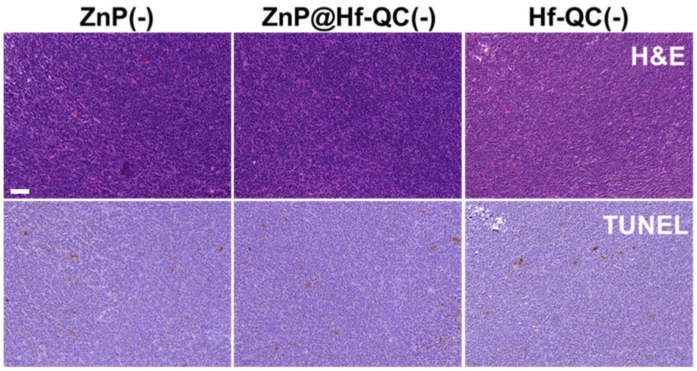

Figure S43. *In vivo* TUNEL staining of excised tumor sections in control groups of CT26 tumors (nuclei stained by hematoxylin in blue; TUNEL positive by DAB in red; scale bars are 100 μm)**.**

S5.3. *In vivo* biodistribution

To evaluate *in vivo* biodistribution of Hf-QC and ZnP@Hf-QC, CT26 tumor-bearing BALB/c mouse model was established by inoculating $2x10^6$ cells/mouse subcutaneously at day 1. At day 7, mice with tumor volumes around 100 mm^3 were intravenously injected with PEGylated Hf-QC or ZnP@Hf-QC with equivalent Hf dose of 0.88 μmol (N=3). After 12 hours, the mice were sacrificed, and blood, tumor, tumor draining lymph node, heart, lung, liver, spleen and kidney were weighed and digested with 99% concentrated HNO_3 and 1% HF. The Hf amount was determined by ICP-MS and Hf ID%/g was used to assess *in vivo* biodistribution of Hf-QC and ZnP@Hf-QC.

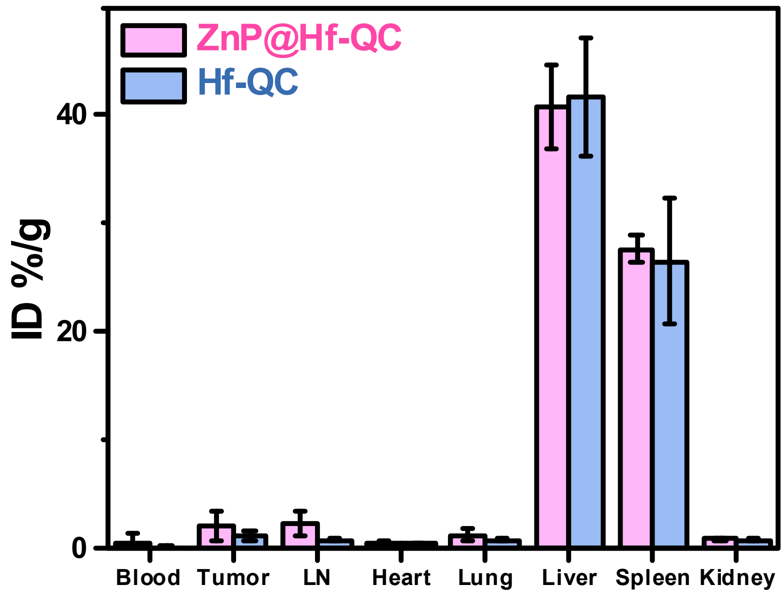

Figure S44. *In vivo* biodistribution of ZnP@Hf-QC and Hf-QC on CT26 tumor-bearing BALB/c mice.

S5.4 Statistical analysis

All the statistical analysis was performed on Origin Lab software by One-way Repeated Measures ANOVA method for *in vivo* efficacy study with Tukey's honest significance test to determine whether the difference between each group was significant. The p values were defined as * $p<0.05$, ** $p<0.01$, *** $p<0.001$, **** $p<0.0001$ in all the figures. The tumor volumes and tumor weights of the last day of experiment were chosen for analysis (N=5).

Table S1. ANOVA analysis and p-values by Tukey test of CT26 tumor volumes at day 22.

**Group**	**Prob>|t|**
PBS(+) v.s. ZnP(-)	0.99796
PBS(+) v.s. ZnP(+)	**0.02633**
PBS(+) v.s. ZnP@Hf-QC(-)	0.75309
PBS(+) v.s. ZnP@Hf-QC(+)	**2.17×10^{-7}**
PBS(+) v.s. Hf-QC(-)	0.99938
PBS(+) v.s. Hf-QC(+)	0.90814
ZnP(-) v.s. ZnP(+)	0.08381
ZnP(-) v.s. ZnP@Hf-QC(-)	0.96096
ZnP(-) v.s. ZnP@Hf-QC(+)	**7.66×10^{-7}**
ZnP(-) v.s. Hf-QC(-)	1
ZnP(-) v.s. Hf-QC(+)	0.99597
ZnP(+) v.s. ZnP@Hf-QC(-)	0.42814
ZnP(+) v.s. ZnP@Hf-QC(+)	**9.19×10^{-4}**
ZnP(+) v.s. Hf-QC(-)	0.06787
ZnP(+) v.s. Hf-QC(+)	0.25855
ZnP@Hf-QC (-) v.s. ZnP@Hf-QC(+)	**6.90×10^{-6}**
ZnP@Hf-QC (-) v.s. Hf-QC(-)	0.93737
ZnP@Hf-QC (-) v.s. Hf-QC(+)	0.99985
ZnP@Hf-QC (+) v.s. Hf-QC(-)	**6.04×10^{-7}**
ZnP@Hf-QC (+) v.s. Hf-QC(+)	**3.13×10^{-6}**
Hf-QC(-) v.s. Hf-QC(+)	0.99074

Table S2. ANOVA analysis and p-values by Tukey test of CT26 tumor weights at day 22.

**Group**	**Prob>|t|**
PBS(+) v.s. ZnP(-)	0.99901
PBS(+) v.s. ZnP(+)	**0.01814**
PBS(+) v.s. ZnP@Hf-QC(-)	0.64394

PBS(+) v.s. ZnP@Hf-QC(+)	**3.23×10^{-6}**
PBS(+) v.s. Hf-QC(-)	0.99979
PBS(+) v.s. Hf-QC(+)	0.93103
ZnP(-) v.s. ZnP(+)	0.05201
ZnP(-) v.s. ZnP@Hf-QC(-)	0.88966
ZnP(-) v.s. ZnP@Hf-QC(+)	**9.74×10^{-6}**
ZnP(-) v.s. Hf-QC(-)	1
ZnP(-) v.s. Hf-QC(+)	0.99626
ZnP(+) v.s. ZnP@Hf-QC(-)	0.44067
ZnP(+) v.s. ZnP@Hf-QC(+)	**0.0218**
ZnP(+) v.s. Hf-QC(-)	0.04071
ZnP(+) v.s. Hf-QC(+)	0.1718
ZnP@Hf-QC(-) v.s. ZnP@Hf-QC(+)	**1.73×10^{-4}**
ZnP@Hf-QC(-) v.s. Hf-QC(-)	0.84221
ZnP@Hf-QC(-) v.s. Hf-QC(+)	0.99635
ZnP@Hf-QC(+) v.s. Hf-QC(-)	**7.47×10^{-6}**
ZnP@Hf-QC(+) v.s. Hf-QC(+)	**4.06×10^{-5}**
Hf-QC(-) v.s. Hf-QC(+)	0.99058

Table S3. ANOVA analysis and p-values by Tukey test of MC38 tumor volumes at day 24.

**Group**	**Prob>|t|**
PBS(+) v.s. ZnP(+)	**0.02687**
PBS(+) v.s. ZnP@Hf-QC(+)	**2.12373×10^{-4}**
PBS(+) v.s. Hf-QC(+)	0.8066
ZnP(+) v.s. ZnP@Hf-QC(+)	**0.0191**
ZnP(+) v.s. Hf-QC(+)	**0.01693**
ZnP@Hf-QC(+) v.s. Hf-QC(+)	**1.41391×10^{-4}**

Table S4. ANOVA analysis and p-values by Tukey test of MC38 tumor weights at day 24.

**Group**	**Prob>|t|**

PBS(+) v.s. ZnP(+)	**0.03362**
PBS(+) v.s. ZnP@Hf-QC(+)	**1.96439×10^{-4}**
PBS(+) v.s. Hf-QC(+)	0.63073
ZnP(+) v.s. ZnP@Hf-QC(+)	**0.01394**
ZnP(+) v.s. Hf-QC(+)	0.081
ZnP@Hf-QC(+) v.s. Hf-QC(+)	**4.47806×10^{-4}**

S6. References

(1) Bankhead, P.; Loughrey, M. B.; Fernández, J. A.; Dombrowski, Y.; McArt, D. G.; Dunne, P. D.; McQuaid, S.; Gray, R. T.; Murray, L. J.; Coleman, H. G.; James, J. A.; Salto-Tellez, M.; Hamilton, P. W., QuPath: Open source software for digital pathology image analysis. *Sci. Rep.* **2017,** *7* (1), 16878.

(2) Nash, G. T.; Luo, T.; Lan, G.; Ni, K.; Kaufmann, M.; Lin, W., Nanoscale Metal–Organic Layer Isolates Phthalocyanines for Efficient Mitochondria-Targeted Photodynamic Therapy. *J. Am. Chem. Soc.* **2021,** *143* (5), 2194-2199.
